# A time-stamping tactile sensor enabled by pseudoconductive interface design at dielectric heterojunctions

**DOI:** 10.1126/sciadv.aec9793

**Published:** 2026-04-22

**Authors:** Byungseok Seo, Dowon Noh, Yong Choi, Yongfa Cheng, Yeonbin Seong, Xinqi Chen, Wonjoon Choi

**Affiliations:** ^1^ The NUANCE Center, Northwestern University, Evanston, IL 60208, USA.; ^2^ School of Mechanical Engineering, Korea University, Seoul 02841, Republic of Korea.; ^3^ Department of Materials Science and Engineering, Northwestern University, Evanston, IL 60208, USA.; ^4^ Department of Mechanical Engineering, Northwestern University, Evanston, IL 60208, USA.

## Abstract

Capturing the spatiotemporal aspects of tactile stimuli is essential for real-time and intelligent operation in emerging human-machine interfaces, electronic skin, and neuromorphic systems. However, most time-resolved tactile sensors rely on complex architectures involving conductive components, active switching elements, or external circuitry, limiting their flexibility and energy efficiency. Here, we introduce a time-stamping tactile sensing strategy on the basis of mechanical stimulus–driven pseudoconductive (MSPC) channels that form at dielectric heterojunctions. Comprehensive band-structure analysis of combinations among 11 dielectric materials reveals that MSPC channels arise from band alignment governed by Fermi-level shifts, quasi-Fermi formation, and field-induced band tilting. The MSPC favorability index is devised to quantitatively predict optimal combinations across 72 dielectric heterojunctions. Mechanical charging activates dielectric pathways that transmit mechanoelectric signals over extended distances, achieving an 854% enhancement across 129 millimeters. A proof-of-concept time-stamping tactile sensor leverages the time-dependent deactivation dynamics of MSPC channels to intrinsically encode spatial and temporal information, offering a passive, scalable, and energy-efficient route for next-generation tactile perception.

## Teaser

Mechanically driven pseudoconductive channels in dielectric interfaces enable passive, bias-free time-stamping tactile sensing.

## INTRODUCTION

Tactile sensing technologies are essential for a broad range of emerging applications, including human-machine interfaces, robotics, prosthetics, electronic skin, and wearable health monitoring systems (*1*–*3*). In these domains, the ability to detect and interpret physical interactions is critical for enabling real-time, adaptive, and intelligent responses (*4*–*7*). At the core of tactile sensing lies the principle of mechanoelectric energy conversion, in which mechanical stimuli such as pressure, deformation, or touch are transduced into measurable electrical signals (*8*–*10*). This principle has enabled the development of various sensing mechanisms, including capacitive, resistive, piezoelectric, and triboelectric sensors, which have substantially advanced in sensitivity, spatial resolution, and compatibility with soft, flexible substrates (*11*–*14*). Although these technologies have proven effective in detecting the position and intensity of mechanical input, they remain largely limited to static or spatial information (*15*, *16*). In dynamic or interactive scenarios, such as gesture recognition, haptic feedback, or sequential control, the order, duration, and timing of contact events carry critical information (*17*–*20*). In such cases, the absence of intrinsic temporal sensing becomes a significant limitation. As systems increasingly shift toward time-aware and event-driven operation, tactile sensors must evolve to capture not only where and what degree a mechanical interaction occurs but also when it occurs.

To address this need, various time-resolved tactile sensing strategies have been explored, including active-matrix arrays with multiplexed addressing, pixel-integrated transistors with dynamic readout circuits, and sensor nodes incorporating memory or timing logic (*21*–*23*). These architectures aim to extract temporal information by storing signal histories or actively controlling signal acquisition sequences (*24*, *25*). However, such approaches typically rely on conductive materials, external biasing, and complex circuitry (*26*–*28*). These requirements introduce several fundamental limitations. Specifically, conductive components often reduce mechanical flexibility and are prone to degradation under repeated deformation. Meanwhile, external power sources increase energy consumption and hinder integration into self-powered systems, and complex circuits impede scalability, particularly in large-area or wearable formats (*29*–*31*). Furthermore, the reliance on memory or timing logic not only consumes additional power but also introduces latency in signal processing that undermines real-time responsiveness (*32*, *33*). In this context, a fundamentally more efficient approach is to directly encode temporal information within the electrical output of the sensor itself without relying on memory, logic circuits, or continuous data acquisition. A sensor capable of intrinsically time-stamping mechanical events at the moment of contact would simplify the system architecture while enhancing responsiveness, energy efficiency, and scalability (*34*). Nevertheless, realizing such functionality using only passive materials, which do not rely on external power, logic circuits, or active components for signal processing, remains a significant challenge that is yet to be overcome (*35*).

Meanwhile, dielectric materials have emerged as promising candidates for tactile sensing, owing to their high mechanoelectric conversion capability, intrinsic flexibility, chemical stability, biocompatibility, and ease of processing (*36*–*38*) Their electrically insulating nature minimizes leakage current, enables low-power operation, and allows for simplified device architectures (*39*–*42*). Accordingly, dielectrics have been widely used in tactile sensing systems, where their passive properties contribute to reliable and durable performance (*43*–*45*). However, this passivity also imposes a key limitation: Conventional dielectrics cannot actively facilitate or sustain charge carrier transport over long distance (*46*–*48*). As such, time-resolved or long-range signal propagation using dielectrics alone remains difficult, as they do not inherently have mechanisms for dynamic signal routing or temporal encoding (*49*, *50*). Fully dielectric sensor platforms have therefore remained largely confined to spatial sensing applications, unable to deliver spatiotemporal functionality without the integration of conductive or active components (*51*, *52*).

In contrast to that of semiconductors, which have benefited from decades of research developing sophisticated interface engineering techniques such as p-n junctions, heterojunctions, and Schottky barriers, the interfacial behavior of dielectric materials remains largely unexplored (*53*–*55*). Although dielectric-dielectric interfaces are often considered electrically inert, in principle, they can support rich electronic phenomena if their energy band structures are properly aligned and perturbed (*56*, *57*). In particular, the ability to manipulate interfacial energy levels in response to external stimuli offers an effective pathway to control charge carrier dynamics without relying on conventional conduction mechanisms (*58*, *59*). Developing a fundamental understanding of such interfaces is therefore essential for unlocking previously unidentified forms of signal transmission and functionality in dielectric devices. By reimagining dielectric junctions not as passive boundaries but as active sites for charge modulation and transport, previously unexplored classes of electronic components that are passive yet highly multifunctional may be realized.

**Fig. 1.**
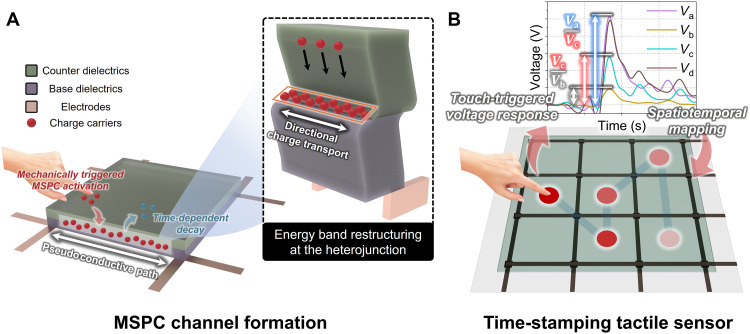
Conceptual schematic of MSPC channel formation and MSPC channel–based time-stamping tactile sensing. (**A**) Mechanically triggered MSPC channel activation at a dielectric heterojunction enabling directional charge transport through a pseudoconductive path, followed by time-dependent deactivation. (**B**) Resulting tactile sensor architecture intrinsically encoding spatiotemporal information in mechanoelectric output signals via voltage ratios without external memory or bias.

Here, we developed a signal transmission strategy on the basis of mechanical stimulus–driven pseudoconductive (MSPC) channels formed at dielectric heterojunctions (Fig. 1A). Conventional tactile sensors typically rely on geometry-dependent changes in capacitance or resistance under externally biased readout or generate signals through bulk polarization or surface electrification in piezoelectric and triboelectric systems. In contrast, an MSPC channel originates from mechanically induced interfacial charge transport at a dielectric-dielectric heterojunction, enabled by energy-band restructuring and quasi-Fermi level redistribution rather than bulk deformation or static surface charging. On the basis of this mechanism, we demonstrate a self-contained time-stamping tactile sensor that could intrinsically encode spatial and temporal information entirely through dielectric interfaces without requiring external memory, timing circuits, or active switching components (Fig. 1B). First, energy band restructuring was examined at various dielectric heterojunctions, which occurred through via Fermi level shifts, quasi-Fermi level formation, and electric field–induced band tilting. Next, a comprehensive framework was established for understanding and using MSPC channels that could actively control charge carrier transport at the dielectric interface. To facilitate predictive material selection, a quantitative metric, the MSPC favorability index (*MFI*), was introduced. The proposed concept was validated through electrical signal transmission tests conducted along solely dielectric pathways, and a proof-of-concept time-stamping tactile sensor architecture was demonstrated using MSPC-active dielectric combinations. This sensor exploited the time-dependent decay of MSPC channels to encode temporal information intrinsically without relying on external bias, memory, or logic circuitry. These findings highlight the potential of MSPC channel–based strategies for enabling energy-efficient, structurally simple, and scalable spatiotemporal sensing platforms, with broad applicability in wearable electronics, electronic skin, soft robotics, neuromorphic systems, and autonomous sensory interfaces.

## RESULTS

### Measuring the energy band of pristine dielectrics

**Fig. 2.**
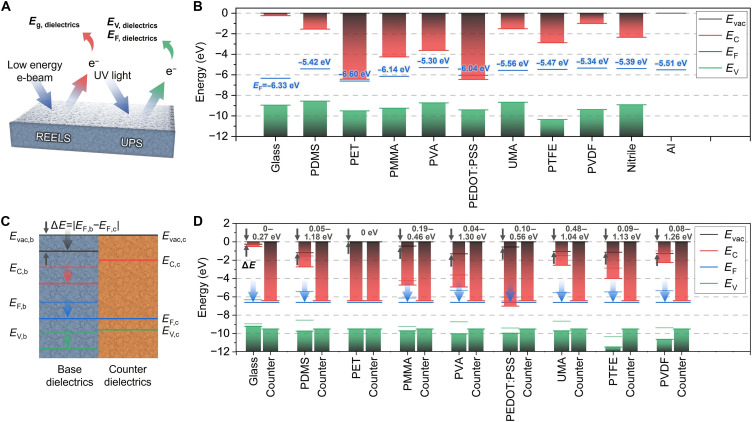
Energy band measurements of individual and combined dielectric materials. (**A**) Measurement of energy bands using REELS for bandgap energy and UPS for determining the VBM and Fermi level. (**B**) Energy band diagrams of individual dielectrics, including glass, PDMS, PET, PMMA, PVA, PEDOT:PSS, UMA, PTFE, PVDF, nitrile, and Al. *E*_vac_, *E*_C_, *E*_F_, and *E*_V_ represent the vacuum level, CBM, Fermi level, and VBM, respectively, while b and c denote the base and counter dielectrics. (**C**) Band alignment at the junction of combined dielectrics composed of base and counter dielectrics. (**D**) Energy band diagrams of combined dielectrics for glass, PDMS, PET, PMMA, PVA, PEDOT:PSS, PTFE, and PVDF with respective counter dielectrics.

Engineering the energy bands at heterojunctions between dielectric materials is crucial for trapping charge carriers and activating MSPC channels along dielectric interfaces. Therefore, the energy bands of various dielectric materials were investigated, including glass, polydimethylsiloxane (PDMS), polyethylene terephthalate (PET), polymethyl methacrylate (PMMA), polyvinyl alcohol (PVA), poly(3,4-ethylenedioxythiophene)-polystyrene sulfonate (PEDOT:PSS), urethane methacrylate (UMA), polytetrafluoroethylene (PTFE), and polyvinylidene fluoride (PVDF). In addition, nitrile and aluminum (Al) were used as the stimulating objects for mechanical stimulus (Fig. 2). As shown in Fig. 2A, the Fermi level (*E*_F_) and valence band maximum (VBM; *E*_V_) of each dielectric material are measured using an ultraviolet photoelectron spectroscopy (UPS) system with a He I excitation source that provided a photon energy of 21.22 eV. High-energy photons from the He I source excited electrons from the material surfaces, which were then emitted as photoelectrons. The energy distribution of these emitted photoelectrons was analyzed to determine two key parameters: the secondary electron (SE) cutoff and the VBM (figs. S1 to S3). The SE cutoff, located at the highest binding energy (*BE*_*max*_) end of the photoelectron spectrum, was identified as the threshold at which electrons just escape from the sample surface. By referencing this SE cutoff position, the Fermi level of each material was precisely determined according to the following equation

$$\phi =h\nu -|{\text{BE}}_{\text{max}}-E_{F}|$$ (1)

where *h*ν is the photon energy, and ϕ is the work function (*60*). These values provide insights into the electronic structure of the materials, with the VBM and Fermi level measurements enabling a detailed understanding of the energy levels. The UPS system was calibrated using metallic (Au) and semiconductor [silicon nitride (SiN*_x_*)] standards to ensure accurate Fermi level and VBM measurements (fig. S4). In addition, to accurately determine the bandgap energies of the dielectric materials, reflected electron energy loss spectroscopy (REELS) was performed (Fig. 2A). In REELS, a low-energy electron beam is directed onto the material surface, where some of these electrons lose energy because of interactions with the electrons in the material. This energy loss, which is characteristic of electronic transitions within the material, provides information about the bandgap (figs. S5 and S6). For reliable results, the REELS setup was calibrated using a known reference material, SiN*_x_*, which enabled the precise alignment of the energy scale (fig. S6). The bandgap values were obtained by fitting the energy loss spectrum and identifying the energy threshold at the onset of electronic transitions. This threshold was then used to calculate the bandgap, yielding accurate and reproducible measurements of the bandgap energies for the dielectric materials. These UPS- and REELS-derived spectroscopic datasets provide a fully quantitative and experimentally grounded basis that serves as the direct evidence framework for validating the MSPC channel–related band restructuring and charge-redistribution behavior, even though the limited probing depth does not allow the direct measurement of the buried dielectric-dielectric interface.

On the basis of the collected information, energy band diagrams were constructed for individual materials (Fig. 2B). The examined dielectric materials included glass, PDMS, PET, PMMA, PVA, PEDOT:PSS, UMA, PTFE, and PVDF, with nitrile and Al serving as stimulating objects to drive the formation of MSPC channels. When these base dielectrics were paired with counter dielectrics, the energy band structures aligned at the heterojunctions of the combined materials (Fig. 2C). The difference in the Fermi levels (∆*E*) between the base and counter dielectrics (*E*_F,b_ and *E*_F,c_, respectively) was calculated as follows

$$∆E=|E_{F,b}-E_{F,c}|$$ (2)

Depending on ∆*E*, the overall energy bands, including the vacuum level (*E*_vac_), conduction band minimum (CBM; *E*_C_), *E*_F_, and VBM (*E*_V_), shifted downward for dielectrics with higher Fermi levels. The extent of this energy band shift varied according to the specific combinations of dielectrics and various counter dielectrics (Fig. 2D), ranging from 0 to 0.27 eV for glass, 0.05 to 1.18 eV for PDMS, 0 eV for PET, 0.19 to 0.46 eV for PMMA, 0.04 to 1.30 eV for PVA, 0.10 to 0.56 eV for PEDOT:PSS, 0.48 to 1.04 eV for UMA, 0.09 to 1.13 eV for PTFE, and 0.08 to 1.26 eV for PVDF. Because of its relatively high individual Fermi level of ~−5.30 eV, PVA could potentially produce the most significant band alignment. In contrast, PET, with the lowest individual Fermi level of ~−6.60 eV, was less likely to achieve band alignment when paired with any counter dielectric. These distinct energy alignments make them promising candidates for designing robust MSPC channels, offering favorable conditions for controlling the energy band configurations.

### Restructuring of the energy band driven by mechanical stimulation

**Fig. 3.**
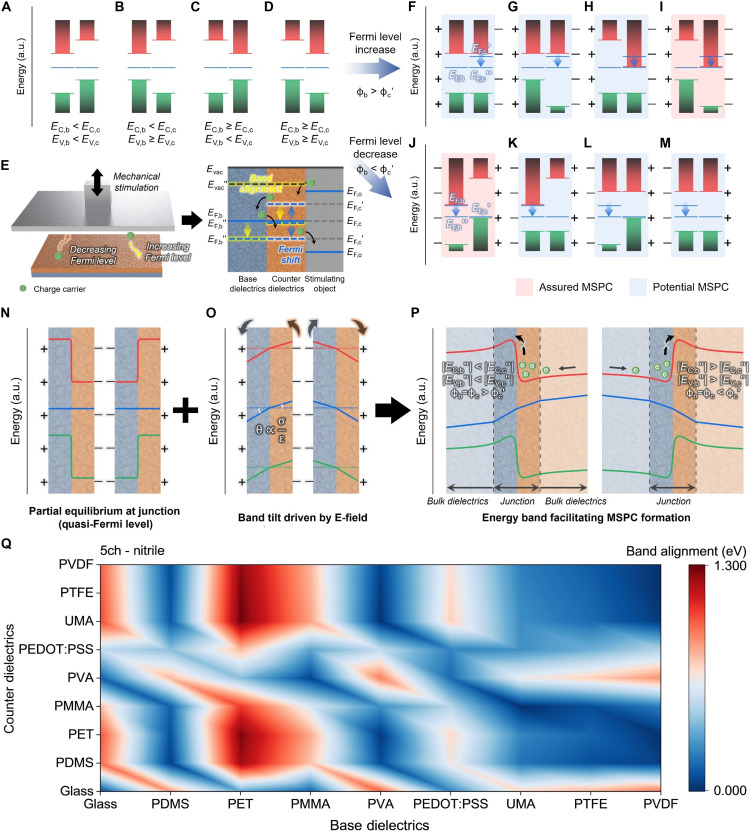
Restructuring of the energy band in combined dielectrics induced by mechanical stimulation. (**A** to **M**) Examination of energy band configurations due to Fermi level shifts before and after mechanical stimulation. (A to D) Energy band configurations of combined dielectrics in their pristine state. a.u., arbitrary units. (E) Mechanism of Fermi level shift induced by mechanical stimuli applied to the combined dielectrics with corresponding band offset adjustments in cases of Fermi level (F to I) increase and (J to M) decrease due to mechanical stimuli. *E*, *E*′, and *E*″ represent the energy levels in the pristine state, immediately after the charging process, and after band alignment is completed, respectively. ϕ and ϕ′ denote the work function in the pristine state and immediately after the charging process, respectively. b, c, and o indicate the base dielectrics, counter dielectrics, and stimulating object, respectively. Additional effects of (**N**) quasi-Fermi level, (**O**) band tilting induced by the electric field, and (**P**) the final restructured energy band necessary for robust MSPC channel formation. (**Q**) Evaluation of band alignment amount after the charging process with stimulating objects of nitrile at a frequency of 2.5 Hz for 5 min.

Next, the energy band conditions for forming pseudoconductive channels through mechanical stimuli were investigated (Fig. 3). Figure 3 (A to D) shows cases of heterojunctions between base and counter dielectrics with different band structures. When a mechanical stimulus is applied to the counter dielectric, charge carriers move according to the Fermi level difference (∆*E*) between the stimulating object (*E*_F,o_) and the counter dielectric (*E*_F,c_), causing a Fermi level shift (Fig. 3E). This, in turn, realigns the bands within both the base and counter dielectrics. We refer to this process of restructuring the energy band, which triggers charge carrier movement, as the charging process. When the Fermi level of the stimulating object (*E*_F,o_) is higher than that of the counter dielectric (*E*_F,c_), the mechanical stimulus immediately increases the Fermi level of the counter dielectric (*E*_F,c_′). Consequently, the work function of the counter dielectric (ϕ_c_′) becomes lower than that of the base dielectric (ϕ_b_) after the charging process, causing charge carriers to migrate from the counter dielectric to the base dielectric. This charge movement creates a negative dielectric field in the counter dielectric and a positive dielectric field in the base dielectric. Once band alignment is completed, the two Fermi levels become identical (*E*_F,b_ = *E*_F,c_″), inducing overall energy level shifts and resulting in various CBM and VBM relations in the heterojunction (Fig. 3, F to I). In contrast, when the charging process decreases the Fermi level of the counter dielectric (*E*_F,c_′), the energy bands align through the opposite mechanism (*E*_F,b_″ = *E*_F,c_′), and positive and negative electric fields form in the counter and base dielectrics, respectively (Fig. 3, J to M).

Once the Fermi levels of the base and counter dielectrics align, the system reaches partial equilibrium at the heterojunction (Fig. 3N). In this state, the quasi-Fermi levels for electrons reflect the redistribution of charge carriers because of the mechanical stimulus during the charging process (*61*, *62*). The alignment of these quasi-Fermi levels between the base and counter dielectrics is crucial for connecting the conduction and valence bands, forming an energy barrier at the junction. The electric fields induced by the mechanical stimulus influence this alignment, facilitating the creation of an energy barrier that governs charge carrier migration at the junction. Furthermore, the electric fields generated by the mechanical stimulus induce energy band tilting at the heterojunction (Fig. 3O). During the charging process, an increase in the Fermi level of the counter dielectric (ϕ_b_ > ϕ_c_′) tilts the band counterclockwise, whereas a decrease (ϕ_b_ < ϕ_c_′) tilts it clockwise (*63*). The extent of this tilting varies according to the relative permittivity of the materials (ε) and the surface charge density (σ), resulting in different tilting angles (θ) for the base and counter dielectrics

$$\theta \propto \frac{\sigma }{\varepsilon }$$ (3)

This differential tilting critically influences the movement of charge carriers toward the junction. Materials with lower relative permittivity exhibit more pronounced band tilting, thereby affecting the charge carrier flow and the formation of energy barriers at the heterojunction.

The final energy structures favorable for forming MSPC channels were derived from these three key mechanisms: band alignment due to Fermi level shifts, quasi-Fermi level adjustment, and energy band tilting driven by the electric fields (Fig. 3P). Unlike that at the junction, the charge distribution in the bulk dielectric regions remains relatively uniform, resulting in gradual changes rather than significant variations. Robust MSPC channels can be formed when specific conditions are met during the restructuring process in the base and counter dielectrics

$$|{E_{C,b}}^{\prime \prime }|<|{E_{C,c}}^{\prime \prime }|,|{E_{V,b}}^{\prime \prime }|<|{E_{V,c}}^{\prime \prime }|,\phi_{b}=\phi_{c}>{\phi_{c}}^{\prime }$$ (4)

$$|{E_{C,b}}^{\prime \prime }|>|{E_{C,c}}^{\prime \prime }|,|{E_{V,b}}^{\prime \prime }|>|{E_{V,c}}^{\prime \prime }|,\phi_{b}=\phi_{c}<{\phi_{c}}^{\prime }$$ (5)

This restructuring is collectively influenced by several parameters, including the CBM, VBM, and Fermi levels of the base and counter dielectrics, as well as the properties of the stimulating object used during the charging process. MSPC channels will form either when the CBM and VBM of the base dielectric is higher than that of the counter dielectric with an increase in the Fermi level or when they are lower with a decrease in the Fermi level (Fig. 3, I and J). Other parameter combinations may also potentially form MSPC channels if conditions in Eq. 4 or 5 are satisfied after the charging process (Fig. 3, F to H and K to M).

To experimentally validate the predicted conditions for MSPC channel formation, mechanical stimuli were applied during the charging process to combined dielectrics using nitrile and Al stimulating objects, which had Fermi levels of −5.39 and −5.51 eV, respectively (Fig. 3Q and fig. S7). Following the charging process at a frequency of 2.5 Hz for 5 min, the resulting Fermi level shifts, which were expected to initiate energy band restructuring and activate the MSPC phenomenon, were evaluated (figs. S8 to S10). The extent of energy band alignment due to Fermi level shifts varied from 0 to 1.30 eV, depending on the energy band structures of the combined dielectrics and the properties of the stimulating objects, ultimately determining the final energy bands for MSPC channel formation. For instance, the glass/PET combination exemplified guaranteed MSPC channel formation (Fig. 3I and fig. S11). In their pristine states, both the CBM and VBM of glass were higher than those of PET. During the charging process, PET received charge carriers from Al, which had a higher Fermi level (*E*_F,o_ > *E*_F,c_), thus increasing the Fermi level of PET (*E*_F,c_′). This reduced the energy in PET through band alignment, amplifying the differences in the CBM and VBM between the two dielectrics and creating highly favorable conditions for MSPC channel formation. Conversely, the PVA/PDMS combination ensured MSPC channel formation through the opposite mechanism following the charging process (*E*_F,o_ < *E*_F,c_), where the decreased Fermi level of PDMS created a favorable environment for forming MSPC channels (Fig. 3J and fig. S11). In addition, other combinations with randomly arranged CBM and VBM could also potentially form MSPC channels (Fig. 3, F to H and K to M). For example, the glass/UMA and PVA/PMMA combinations exhibited increased and decreased Fermi levels, respectively, after the charging process (fig. S11). This energy band restructuring facilitated conditions conducive to MSPC channel formation. These results demonstrate that MSPC channel formation at the heterojunction can be influenced by the energy relationship between different dielectrics, as well as the electronic properties of the dielectrics and the stimulating object. This implies that comprehensively considering these factors can guide the design of energy structures conducive to forming robust MSPC channels. Notably, even simply touching the device with a nitrile-gloved finger could significantly shift the Fermi level, causing energy band restructuring and effectively activating an MSPC channel (figs. S12 and S13). This finding validates the strong prospects of MSPC technology for wearable device applications.

### Evaluation of the *MFI*

**Fig. 4.**
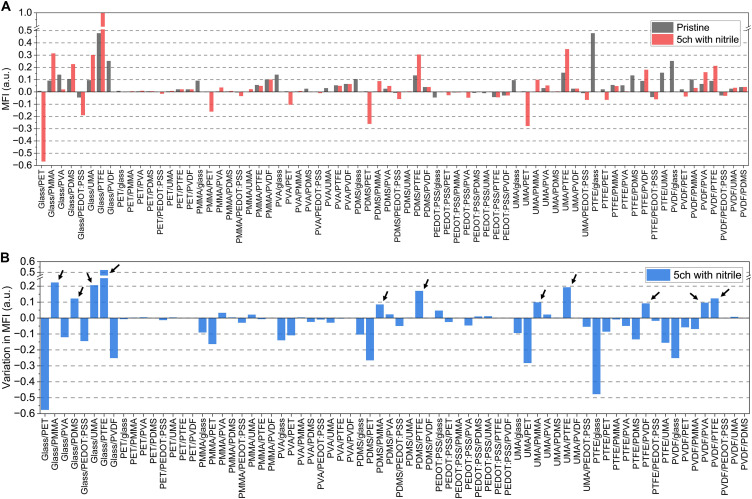
Evaluation of the *MFI* for combined dielectrics. (**A**) *MFI* of combined dielectrics consisting of base and counter dielectrics in their pristine state and after a 5-min charging process with nitrile, considering variations in the CBM, VBM, charge affinity (χ), work function (ϕ), and band tilting propensity (*P*_BT_). (**B**) Comparison of the *MFI* variations after the charging process with nitrile relative to the pristine value, highlighting combined dielectrics with significant changes indicated by arrows.

Given that different material combinations may activate the MSPC phenomenon with various degrees of certainty, an index termed the *MFI* was established for quantifying how favorable conditions are for forming MSPC channels. This index considers variations in the CBM, VBM, charge affinity (χ), work function (ϕ), and band tilting direction (Fig. 4 and fig. S14). Through the stepwise mechanisms of energy band restructuring, large differences in the CBM (*E*_C,c_″ − *E*_C,b_″) and VBM (*E*_V,c_″ − *E*_V,b_″) across two dielectrics after the charging process were identified as favorable for MSPC channel activation (Fig. 3, A to P). In addition, when the Fermi level is positioned further from both the CBM and VBM, charge carriers are more likely to be trapped at the heterojunction rather than escaping. In most cases, lowering the Fermi level below the VBM requires excessive energy input, which is difficult to achieve through mechanical stimuli alone. Therefore, only cases where the Fermi level rises above the CBM are considered sufficient for MSPC channels. This criterion can be expressed as the difference between the CBM and the Fermi level (*E*_C_″ − *E*_F_″) or as the difference between the work function and the charge affinity (ϕ″ − χ″). When the work function is smaller than the charge affinity (ϕ″ − χ″ < 0), the Fermi level overlaps with the conduction band (*E*_C_″ − *E*_F_″ < 0), preventing charge carrier trapping at the heterojunction, resulting in a negative *MFI*. It is also necessary to evaluate whether the direction of band tilting induced by mechanical stimuli corresponds to the CBM and VBM relationship between the two dielectrics. This factor, termed the band tilting propensity (*P*_BT_), was assigned values of 2, 1, or 0 on the basis of specific conditions. Incorporating the *P*_BT_ and the rest of the terms described above, the equation for the MSPC favorability coefficient (*MFC*) was thus established as follows

$$\begin{array}{cc} \text{MFC} & =|∆\text{CBM}|\times |∆\text{VBM}|\times \text{min}\left[{E_{C,c}}^{\prime \prime }-{E_{F}}^{\prime \prime },{E_{C,b}}^{\prime \prime }-{E_{F}}^{\prime \prime }\right]\times P_{\text{BT}}\\ & =|{E_{C,c}}^{\prime \prime }-{E_{C,b}}^{\prime \prime }|\times |{E_{V,c}}^{\prime \prime }-{E_{V,b}}^{\prime \prime }|\times \text{min}\left[\phi^{\prime \prime }-{\chi_{c}}^{\prime \prime },\phi^{\prime \prime }-{\chi_{b}}^{\prime \prime }\right]\times P_{\text{BT}} \end{array}$$ (6)

where

$$P_{\text{BT}}=\left\{ \begin{array}{c} 2,\;\text{if}\;\phi_{b}>{\phi_{c}}^{\prime }\;\text{and}\;{E_{C,b}}^{\prime \prime }>{E_{C,c}}^{\prime \prime }\;\text{and}\;{E_{V,b}}^{\prime \prime }>{E_{V,c}}^{\prime \prime }\\ \text{or}\;\phi_{b}<{\phi_{c}}^{\prime }\;\text{and}\;{E_{C,b}}^{\prime \prime }<{E_{C,c}}^{\prime \prime }\;\text{and}\;{E_{V,b}}^{\prime \prime }<{E_{V,c}}^{\prime \prime }\\ 1,\text{if}\;\left({E_{C,c}}^{\prime \prime }-{E_{C,b}}^{\prime \prime }\right)\times \left({E_{V,c}}^{\prime \prime }-{E_{V,b}}^{\prime \prime }\right)<0\\ 0,\text{otherwise} \end{array}\right.$$ (7)

The final *MFI* was derived by normalizing the *MFC* with its maximum value as follows

$$\text{MFI}=\frac{\text{MFC}}{{\text{MFC}}_{\text{max}}}$$ (8)

These parameters were used to quantitatively analyze the conditions for MSPC channel formation (Fig. 4). Notably, combinations of materials in their pristine states, such as glass with PTFE, PVDF with glass, PTFE with UMA, PVA with glass, and PTFE with PDMS, exhibited high *MFI* values (Fig. 4A). However, after applying the charging process with an Al or nitrile object, the *MFI* varied significantly across dielectric combinations. For instance, the *MFI* of the glass/PTFE combination increased from 0.48 in the pristine state to 1.00 and 0.96 after charging with nitrile and Al, respectively, representing enhancements of 208 and 200%, indicating the optimal conditions for MSPC channel activation. Conversely, when the materials were arranged in the reverse PTFE/glass configuration, the energy band structure and electric field orientation were also reversed, resulting in a *P*_BT_ value of 0, which did not meet the conditions necessary for forming MSPC channels. Combinations such as glass/PVDF, PVDF/glass, PTFE/UMA, glass/PVA, and PVA/glass failed to satisfy certain parameters after the charging process, preventing successful MSPC channel formation. Only certain combinations, namely, UMA/PTFE, PTFE/PDMS, and PDMS/PTFE, showed *MFI* improvements up to 228%, forming favorable conditions for MSPC channels.

These results demonstrate that MSPC channel activation requires careful consideration of multiple parameters, as the charging process can substantially alter the energy band structure and *MFI*. The changes in *MFI* before and after the charging process are summarized in Fig. 4B. Material combinations with high variations in the *MFI* exceeding the threshold of 0.085, such as glass/PMMA, glass/PDMS, glass/UMA, glass/PTFE, PDMS/PMMA, PDMS/PTFE, UMA/PMMA, UMA/PTFE, PTFE/PVDF, PVDF/PVA, and PVDF/PTFE, can be considered candidate pairs that can form pseudoconductive channels through simple mechanical stimulation (figs. S15 to S17). These materials demonstrate good prospects for future MSPC channel–based sensor technologies, pending further evaluation of their electrical properties while considering fabrication feasibility and interfacial compatibility. Moreover, the ability to activate MSPC channels through a nitrile-gloved finger further highlights the significant promise of the MSPC phenomenon for wearable device applications (figs. S18 to S20). In particular, the fact that different stimulating materials modulate the *MFI* adds another controllable parameter that may be useful for the future design of material-responsive MSPC systems.

### Electrical signal transmission tests using MSPC devices

**Fig. 5.**
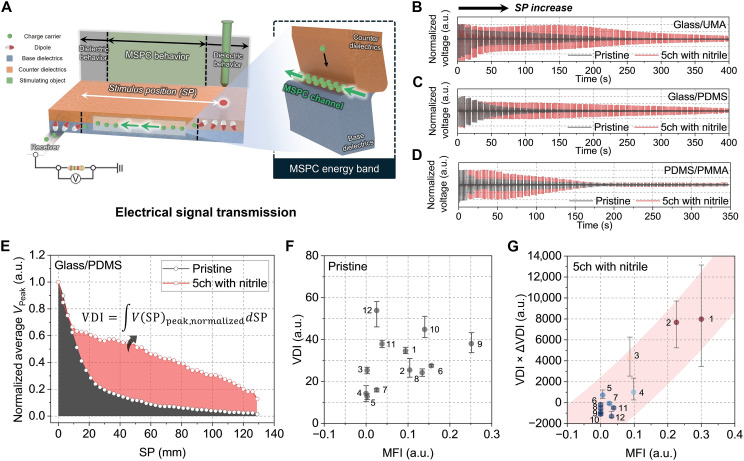
Electrical signal transmission test using MSPC devices. (**A**) Schematic illustration of electrical signal transmission leveraging dielectric and MSPC behaviors across the devices. Normalized voltage profiles of (**B**) glass/UMA, (**C**) glass/PDMS, and (**D**) PDMS/PMMA devices as the *SP* increases, generated through mechanoelectric energy conversion. (**E**) Normalized average voltage peak profiles of the glass/PDMS device as a function of *SP*, measured before and after a 5-minute charging process with nitrile. (**F**) Relationship between *MFI* and *VDI*. (**G**) Relationship between *MFI* and *VDI* × Δ*VDI* variation for MSPC devices, including (1) glass/UMA, (2) glass/PDMS, (3) PDMS/PMMA, (4) UMA/PMMA, (5) PMMA/PDMS, (6) PTFE/UMA, (7) UMA/PVDF, (8) PTFE/PDMS, (9) glass/PVDF, (10) PVA/glass, (11) PDMS/PVDF, and (12) PVDF/UMA.

Electrical signal transmission tests were conducted to validate the effectiveness of the MSPC channels (Fig. 5). The test devices were composed of base and counter dielectric layers, with an Al electrode attached beneath the base dielectric at one end of the device to serve as the signal receiver (Fig. 5A). Mechanical stimulation was applied at a location distant from the electrode, and this distance from the electrode to the stimulation point was defined as the stimulus position (*SP*). During the process, mechanical energy was converted to electrical energy via the charge exchange between the stimulating object and the dielectric layers of the device, and the generated signal was transmitted to the receiver. This transmission occurred through both the typical dielectric behavior at both ends of the device and the previously unexplored MSPC behavior. In conventional mechanoelectric conversion devices, electrical signals are transmitted solely through dielectric behavior, and signals significantly dissipate as the *SP* increases. In contrast, devices with a robust MSPC channel formed at the heterojunction can efficiently transmit electrical signals with minimal dissipation. The efficiency of this transmission increases with the degree of MSPC channel activation, producing markedly reduced dissipation compared with pristine dielectric behavior. Therefore, electrical signals can be delivered across the interface with minimal loss, even over extended distances, enabling effective mechanoelectric signal transport using an architecture composed entirely of dielectric materials.

By considering the *MFI* variation and material compatibility, the final MSPC devices under test (DUTs) were prepared, including glass/UMA, glass/PDMS, PDMS/PMMA, UMA/PMMA, PMMA/PDMS, PTFE/UMA, UMA/PVDF, PTFE/PDMS, glass/PVDF, PVA/glass, PDMS/PVDF, and PVDF/UMA. The normalized temporal voltage profiles were measured for various *SP*s ranging from 0 to 129 mm (Fig. 5, B to D, and figs. S21 and S22) as follows

$$V{\left(\text{SP}\right)}_{\text{normalized}}=\frac{V\left(\text{SP}\right)-V{\left(\text{SP}\right)}_{\text{min}}}{V{\left(\text{SP}\right)}_{\text{max}}-V{\left(\text{SP}\right)}_{\text{min}}}$$ (9)

In the pristine state, where the MSPC effect was inactive, DUTs such as glass/UMA, glass/PDMS, and PDMS/PMMA exhibited a sharp voltage drop as the *SP* increased. However, after activating the MSPC channel through a 5-min charging process with nitrile, the voltage was effectively sustained across the *SP* range. For instance, the glass/UMA DUT exhibited an 854% increase in voltage at an *SP* of 90 mm, whereas the glass/PDMS and PDMS/PMMA DUTs showed enhancements of 813% at 111 mm and 164% at 9 mm, respectively. This indicates that robust MSPC channels made electrical signal transmission more efficient, significantly reducing dissipation compared with that attained with conventional dielectric behavior. In addition, the average voltage peak profiles of MSPC devices in both the pristine and charged states were analyzed as a function of the *SP* (Fig. 5E and figs. S23 and S24). A higher integral value indicates better voltage sustainment, providing a quantitative measure of MSPC channel activation. On the basis of this principle, the voltage drop integral (*VDI*) was established to assess the effectiveness of MSPC channel–induced electrical signal transmission, defined as follows

$$\text{VDI}=\int_{0}^{129}V{\left(\text{SP}\right)}_{\text{peak},\text{normalized}}d\text{SP}$$ (10)

External factors such as temperature, humidity, and applied pressure can alter the electrical response of dielectric-based devices by modifying the charge dissipation and the effective contact area at the interface. In contrast, the MSPC phenomenon in our system originates from interfacial processes between the contacting dielectrics and is therefore largely insensitive to these external perturbations. Accordingly, the MSPC channel–enabled voltage sustainment is consistently preserved across the tested environmental and loading conditions (note S1 and figs. S25 to S27). The relationship between the *MFI* and *VDI* in both pristine and charged states of MSPC devices was evaluated (Fig. 5F and figs. S28 and S29). In the pristine state, even if the energy band structure was favorable for MSPC channel formation and resulted in a high *MFI*, the lack of charge carrier supply through mechanical stimulation prevented the activation of the MSPC channel. Consequently, no clear correlation was observed between the *MFI* and *VDI*. In contrast, after a 5-min charging process, the *VDI* became more linearly distributed with respect to the *MFI* (figs. S28 and S29). Therefore, when sufficient charge carriers were supplied to an energy band structure favorable for MSPC channel formation, the channel was successfully activated, enhancing electrical signal transmission. Notably, the *VDI* of devices composed of glass/UMA, glass/PDMS, and PDMS/PMMA dielectric heterojunctions in the charged state increased by 110, 131, and 90%, respectively, with respect to those in their pristine states. As variations in the *VDI* before and after charging play a crucial role in optimizing the performance of the MSPC channels, both the *VDI* values and their variations were considered in assessing the final device performance (Fig. 5G)

$$∆\text{VDI}\left(\%\right)=\frac{{\text{VDI}}_{\text{charged}}-{\text{VDI}}_{\text{pristine}}}{{\text{VDI}}_{\text{pristine}}}\times 100$$ (11)

The highest-performing device combinations were glass/UMA with a VDI×∆VDI of 7972 and *MFI* of 0.30, glass/PDMS with a VDI×∆VDI of 7659 and *MFI* of 0.23, and PDMS/PMMA with a VDI×∆VDI of 4283 and *MFI* of 0.09. These results indicate that devices with a well-balanced energy band structure and sufficient charge carrier supply exhibited superior MSPC channel activation, which significantly enhanced the electrical signal transmission efficiency. The strong correlation between *VDI* and *MFI* after charging further supports the importance of optimizing the dielectric material selection and charging process to achieve robust MSPC performance. These findings corroborate the prospects of MSPC channel–based devices for future practical applications requiring efficient and stable mechanoelectric signal transmission.

### Time-stamping tactile sensor applications

**Fig. 6.**
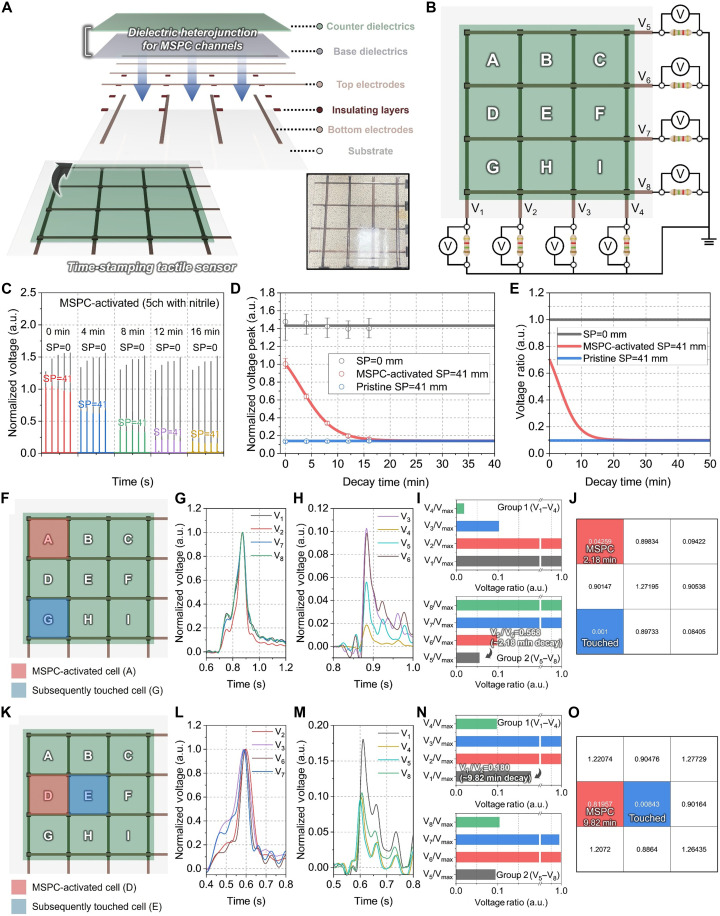
Proof-of-concept demonstration of time-stamping tactile sensing using MSPC channel–based devices. (**A**) Structural design of the time-stamping tactile sensor array, in which each cell comprises dielectric heterojunctions and electrodes that form MSPC channels to generate time-dependent voltage signals in response to localized touch events. (**B**) Electrical circuit diagram consisting of *V*_*1*_ to *V*_*8*_ electrodes and external resistors. (**C**) Time-dependent normalized voltage profiles of MSPC-activated cells (5ch with nitrile) at *SP* = 0 and *SP* = 41 mm, recorded at various decay times from 0 to 16 min. (**D**) Normalized voltage peak decay curves with Boltzmann and linear fitting at *SP* = 0, MSPC-activated *SP* = 41 mm, and pristine *SP* = 41 mm. (**E**) Voltage ratio of the fitted profiles normalized to the *SP* = 0 mm response. (**F**) Scenario in which the MSPC channel is activated at cell A, followed by a touch at cell G after 2 min. Corresponding normalized voltage profiles for (**G**) group 1 (*V*_*1*_, *V*_*2*_, *V*_*7*_, and *V*_*8*_) and (**H**) group 2 (*V*_*3*_, *V*_*4*_, *V*_*5*_, and *V*_*6*_). (**I**) Voltage ratios for each electrode normalized by the maximum value within each group. (**J**) Spatial mapping of the voltage asymmetry metric *M*, indicating MSPC activation at cell A and subsequent touch at cell G. (**K**) Scenario where the MSPC channel is activated at cell D, followed by a touch at cell E after 10 min. Normalized voltage profiles for (**L**) group 1 (*V*_*2*_, *V*_*3*_, *V*_*6*_, and *V*_*7*_) and (**M**) group 2 (*V*_*1*_, *V*_*4*_, *V*_*5*_, and *V*_*8*_). (**N**) Voltage ratios normalized by maximum values in each group. (**O**) Spatial mapping of the voltage asymmetry metric *M*, indicating MSPC activation at cell D and subsequent touch at cell E.

Leveraging MSPC channels with robust mechanoelectric signal transmission, a time-stamping tactile sensor was developed (Fig. 6). The device was fabricated by stacking orthogonally aligned bottom and top electrode arrays, separated by insulating layers to prevent signal interference, along with base and counter dielectrics that formed dielectric heterojunctions to activate MSPC channels (Fig. 6A and fig. S30). The maximum spacing between the aligned electrodes was determined to be 101.0, 83.2, and 41.8 mm for the glass/UMA, glass/PDMS, and PDMS/PMMA configurations, respectively, all of which resulted in a signal dissipation of 36.8% (fig. S31). To safely guarantee MSPC channel formation across all three top-performing dielectric combinations, the electrode spacing was fixed at 41 mm. Each unit cell defined by the electrode pattern was labeled from A to I (Fig. 6B). Each electrode was connected to a 10-MΩ external resistor and a voltmeter, and the corresponding output voltages were denoted as V_1_ to V_8_. This configuration enabled time-stamped and spatially resolved detection of mechanoelectric signals. Operational durability is further verified by applying repetitive stimulation for 1000 cycles, which yields stable peak responses without noticeable degradation (fig. S32A). Notably, the temporal characteristics are also maintained during cycling, as the response time and recovery time extracted from the voltage peaks remain nearly constant from the initial to final cycles, as shown in fig. S32 (B and C). In addition, these temporal metrics are insensitive to variations in the applied pressure, with both the response time and recovery time remaining essentially unchanged across the tested pressure range, as shown in fig. S33. In this architecture, spatial information is extracted from the deterministic voltage attenuation associated with the *SP*, where adjacent electrodes show the highest response and more distant electrodes exhibit characteristic dissipation. Temporal information is independently obtained from the calibrated decay trajectory of MSPC channels, allowing the elapsed time after prior activation to be inferred from voltage ratios along pristine and MSPC-active pathways. These two decoding steps operate independently, enabling simultaneous resolution of touch location and timing within the same distributed voltage pattern.

To validate the time-stamping capability, the temporal decay behavior of the MSPC channel was first investigated (Fig. 6C). Voltage profiles were measured over time by mechanically touching MSPC-activated cells at two different *SP*s of 0 and 41 mm between the touch point and the electrode. These two *SP*s were used to compare signals captured at the electrodes directly adjacent to the touched cell and at electrodes further away. Under the *SP* = 0 mm condition, the voltage signal was measured at the adjacent electrodes without MSPC channel–mediated transmission along the dielectric interface, regardless of whether the MSPC channel was present. Consequently, the voltage remained nearly constant, with an average value of 1.43 and negligible decay over 16 min. In contrast, when *SP* = 41 mm, signal transmission relies on the MSPC channel–mediated pathway, and the normalized voltage gradually decreased from 1.00 to 0.160 as the decay time increased, indicating time-dependent degradation of the MSPC channel. The extracted voltage peaks were then analyzed across the decay times (Fig. 6D and fig. S34). The *SP* = 0 mm and pristine *SP* = 41 mm cases, which showed no significant changes over time, were fitted with linear functions. In contrast, the MSPC-activated *SP* = 41 mm case exhibited a nonlinear decay and was fitted using the following Boltzmann function

$$V_{\text{normalized peak}}=A_{2}+\frac{A_{1}-A_{2}}{1+\text{exp}\left(\frac{t-t_{0}}{\mathrm{dt}}\right)}$$ (12)

where *t* is the decay time after MSPC activation, and *A*_1_ and *A*_2_ represent the upper and lower bounds of the normalized voltage response, respectively, corresponding to the fully developed and fully dissipated states of the MSPC channel. Specifically, *A*_1_ reflects the potential capacity of a fully charged MSPC pathway, i.e., the maximum voltage response achievable under sustained mechanical charging, while *A*_2_ indicates the residual voltage after complete signal decay. The parameter *t*_0_ denotes the inflection point at which the voltage response reaches its midpoint, and *dt* defines the steepness of the temporal decay. The fitting yielded values of *A*_1_ = 1.33, *A*_2_ = 0.141, *t*_0_ = 2.99 min, and *dt* = 3.09 min. These results demonstrate that the normalized voltage peak decays with high temporal regularity, enabling a precise inference of the time on the basis of the MSPC channel activation state. Moreover, by using optimized readout circuitry, improved electrode shielding, and temporal filtering strategies, the signal-to-noise characteristics can be further enhanced, thereby improving the accuracy of the extracted temporal decay profiles (note S2). To provide a normalized and comparable metric, all voltage peaks were divided by the peak voltage at *SP* = 0 mm, yielding voltage ratios (Fig. 6E). The *SP* = 0 mm and pristine *SP* = 41 mm conditions maintained nearly constant voltage ratios of 1.00 and 0.0960, respectively. Meanwhile, the MSPC-activated *SP* = 41 mm condition exhibited a decreasing trend from 0.700 to 0.110 over 16 min. These observations imply that when a particular cell is touched, electrodes directly adjacent to the touched cell will exhibit behavior similar to the *SP* = 0 mm condition. By contrast, the response of electrodes further away across an MSPC-active dielectric path will reflect the decaying MSPC-activated *SP* = 41 mm response. However, if the cell is not MSPC channel activated, the remote electrode response will follow the pristine *SP* = 41 mm trend. Similarly, at positions two cells away (~85 mm), the resulting voltage ratio will represent a multiplicative combination of the transmission states of the intermediate cells. This spatial attenuation behavior strengthens the principle of decoding both spatial and temporal information from the distributed voltage pattern.

On the basis of this principle, the spatiotemporal sensing resolution of the device was evaluated (Fig. 6, F to O). A scenario was tested in which the MSPC channel was activated at cell A, followed by touching cell G after 2 min (Fig. 6F). The adjacent electrodes surrounding G (*V*_*1*_, *V*_*2*_, *V*_*7*_, and *V*_*8*_) exhibited normalized voltage peaks close to 1.00, indicating direct detection via the shortest dielectric path (*SP* = 0 mm) (Fig. 6G). In contrast, the electrodes *V*_*3*_ and *V*_*6*_, located one cell away from G (41 mm), showed attenuated signals of 0.103 and 0.0985, respectively, consistent with the decay behavior of pristine cells (Fig. 6H). At *V*_*4*_, two cells away from G (~85 mm), the peak voltage was 0.0186, closely matching the squared ratio of the pristine *SP* = 41 mm response, indicating signal transmission through two pristine dielectric paths. V_5_, which was also two cells away from G but whose signal path included the MSPC-activated cell A, exhibited a significantly higher peak of 0.0559. The ratio between *V*_*5*_ and adjacent *V*_*6*_ was 0.568, which corresponded to a decay time of 2.18 min on the basis of the Boltzmann calibration (Fig. 6E).

To quantify the directional signal propagation, the voltages were normalized within each group, group 1 (horizontal: *V*_*1*_ to *V*_*4*_) and group 2 (vertical: *V*_*8*_ to *V*_*5*_), to yield relative voltage ratios (Fig. 6I). In group 1, the ratios decreased from *V*_*1*_/*V*_max_ to *V*_*4*_/*V*_max_ with values of 1.00 to 0.0186, confirming that cells H and I were pristine. In group 2, the *V*_*8*_-to-*V*_*5*_ path produced ratios of 1.00, 1.00, 0.0985, and 0.0559, indicating that cell D was pristine, while cell A was MSPC channel activated ~2.18 min earlier. To spatially map this behavior, a voltage asymmetry metric (*M*) was calculated for each cell on the basis of the differences in voltage ratios across opposing electrode pairs (Fig. 6J)

$$M=\sqrt{{\left(\frac{V_{L}}{V_{\text{max}}}-\frac{V_{R}}{V_{\text{max}}}\right)}^{2}+{\left(\frac{V_{T}}{V_{\text{max}}}-\frac{V_{B}}{V_{\text{max}}}\right)}^{2}}$$ (13)

where *V*_L_, *V*_R_, *V*_T_, and *V*_B_ are the normalized voltages of the left, right, top, and bottom electrodes surrounding each cell, respectively, and *V*_max_ is the maximum voltage in the respective group. Cells that were touched directly exhibited a symmetrical voltage distribution, resulting in near-zero *M* values. In contrast, MSPC-activated cells demonstrated locally elevated voltages relative to neighboring pristine cells, leading to *M* values that were distinctly lower than the pristine background. In this scenario, cell G was identified as the touched cell with *M* = 0.001 and cell A as the MSPC-activated cell with *M* = 0.04259. The estimated decay time error was only 0.18 min (9.00%), confirming high-resolution spatiotemporal sensing.

Another test scenario involved MSPC channel activation at cell D, followed by touching cell E after 10 min (Fig. 6K). As expected, the surrounding electrodes *V*_*2*_, *V*_*3*_, *V*_*6*_, and *V*_*7*_ showed voltage peaks near 1.00, while pristine-path electrodes *V*_*4*_, *V*_*5*_, and *V*_*8*_, each positioned at *SP* = 41 mm from E, exhibited peaks of ~0.0960 (Fig. 6, L and M). In contrast, *V*_*1*_, which lays along the MSPC-activated D path, recorded a higher peak of 0.180. The resulting *V*_*1*_/*V*_*2*_ ratio (~0.180) corresponds to an MSPC decay time of ~9.82 min (Fig. 6N). Voltage asymmetry mapping again confirmed these results, with cell E showing a minimal *M* = 0.00843 that identified it as the touched cell, whereas cell D presented a significantly larger *M* = 0.81957, confirming its MSPC-activated state (Fig. 6O). The estimated decay time error was only 1.80%, indicating reliable time-delay estimation. To strengthen the reliability of the error analysis, two additional activation-touch scenarios were conducted and are summarized in note S4. Specifically, MSPC channel activation at cell E followed by a touch at cell H yielded a time error of 1.80%, while MSPC channel activation at cell B followed by a touch at cell E resulted in a time error of 11.00%. These additional tests reinforce the robustness of the proposed tactile sensing strategy. In addition, the temporal resolution of the device was evaluated by measuring the intrinsic response and recovery times, which were <0.705 and <0.015 s, respectively (fig. S35). Notably, the response time is strongly dependent on the approaching velocity of the stimulating object and can be substantially shorter under faster approach conditions. These measurements provide a baseline for the dynamic behavior of MSPC channel–mediated signal transmission and clarify the time window within which consecutive tactile events can be distinguished. When compared with representative conventional tactile sensor mechanisms, MSPC channel–based tactile sensing uniquely enables intrinsic spatiotemporal information encoding while maintaining practical response times and robust cycling durability.

Collectively, these results demonstrate that MSPC channels enable accurate and memory-free encoding of spatial and temporal information from mechanical stimuli. By embedding touch location and elapsed time directly into a single voltage response, this system captures volatile temporal information without the need for integrated memory or active components. The unique combination of spatial resolution and time sensitivity opens previously unexplored possibilities for secure tactile interfaces, where both contact patterns and timing serve as dual-layer authentication inputs. In addition, the parameters that modulate MSPC behavior, such as the specific pairing of base and counter dielectrics and the material-dependent activation induced by different stimulating objects, provide additional distinguishable features that can be leveraged to further strengthen the security of MSPC channel–enabled tactile interfaces. Given this security-oriented design philosophy, the present architecture is optimized for single-touch events. Nonetheless, the underlying spatiotemporal encoding mechanism provides a promising basis for future extensions toward multitouch sensing in more complex interaction scenarios (note S3).

Moving beyond secure tactile interfaces, the intrinsic capacity of MSPC channels for single-mechanism, power-free spatiotemporal encoding underscores their versatility across a wide range of advanced engineering applications (fig. S37 and note S5). MSPC channel–based sensing can support wearable, epidermal, and soft robotic systems that require lightweight and conformal event detection on deformable surfaces. It also offers potential for electronic-skin platforms used in robotics and prosthetics, where distributed sensing must be achieved without dense wiring or integrated memory. In neuromorphic or event-driven architectures, the volatile temporal decay of MSPC channels may serve as a physical analog of short-term synaptic responses. Furthermore, MSPC channel–driven time-stamping can benefit autonomous or battery-free sensory interfaces in which local mechanical interactions must be recorded without external bias or computation. Collectively, these application opportunities highlight the broader technological relevance of MSPC channel–based spatiotemporal sensing and point toward future development of practical, low-power intelligent surfaces.

## DISCUSSION

In this study, we introduced an innovative concept of MSPC channels formed at dielectric heterojunctions. By systematically investigating the energy band structures of 11 materials, we identified the key conditions that enable the formation of robust MSPC channels, including band alignment via Fermi level shifts, quasi-Fermi level adjustments, and electric field–induced band tilting. The Fermi level shifts reached up to 1.30 eV, enabling substantial band restructuring at the dielectric interfaces. These effects were quantitatively evaluated using the *MFI*, allowing the predictive assessment of 72 different dielectric combinations. Electrical signal transmission tests validated the role of MSPC channels in enhancing signal delivery solely through dielectric interfaces without the need for any conductive layers. Notably, mechanoelectric output signals were transmitted across distances up to 129 mm, with a voltage enhancement of up to 854%, demonstrating the capability of MSPC channels to efficiently deliver signals over a long range. Furthermore, MSPC channel activation increased the *VDI* by up to 131%, and the postactivation *VDI* values exhibited a strong linear correlation with *MFI*, confirming the predictive capability of the energy band–based analysis. On the basis of this phenomenon, a proof-of-concept time-stamping tactile sensor was developed by integrating orthogonally aligned electrode arrays with MSPC-active dielectric pairs. By leveraging the time-dependent decay of MSPC channels, the sensor successfully distinguished both the location and timing of tactile events within a 16-min time window, achieving spatiotemporal resolution with less than 0.18 min (11.00% error). This capability highlights the reliability and precision of the MSPC channel–based architecture for time-resolved tactile sensing without the need for memory or active components. To our knowledge, this study established a first-of-its-kind design strategy for fully dielectric, energy-efficient, and bias-free spatiotemporal sensors, offering excellent prospects for future applications in wearable electronics, robotics, and human-machine interfaces.

## MATERIALS AND METHODS

### Material and test device preparation

The following dielectric materials were prepared: glass, PET, PTFE, PDMS, PVA, PMMA, PVDF, PEDOT:PSS, and UMA. Glass, PET, and PTFE were used as solid substrates, while PDMS, PVA, PMMA, PVDF, and UMA were solution processed and cured into freestanding films with a thickness of 1 mm. The glass substrates, PET films, and PTFE sheets were cut into 20- by 10-cm pieces and cleaned with deionized water and ethanol. PDMS was prepared by mixing base and curing agent (SYLGARD 184, Sigma-Aldrich) at a 10:1 ratio (w/w). The mixture was coated onto glass substrates at 5 mm/s using a knife coater and cured at 80°C for 4 hours. PVA was prepared by dissolving PVA powder [weight-average molecular weight (*M*_w_) = 89,000 to 98,000, Sigma-Aldrich] in dimethyl sulfoxide at 10% (w/v), stirred at 80°C for 3 hours, coated onto glass, and dried at 40°C for 24 hours. PMMA was fabricated by dissolving PMMA powder (*M*_w_ ~ 350,000, Sigma-Aldrich) in toluene at 10% (w/v), stirred at 80°C for 4 hours, coated onto glass at 3 mm/s, and dried at 40°C for 24 hours. PVDF was prepared by dissolving PVDF powder (*M*_w_ ~ 534,000, Sigma-Aldrich) in dimethylformamide at 15% (w/v), stirred at 80°C for 2 hours, and coated onto glass. A PEDOT:PSS solution (0.8 wt % in water, Sigma-Aldrich) was cast onto glass and dried in an oven at 80°C for 6 hours. UMA films were fabricated using photopolymer resin (Clear Resin v4, Formlabs), coated onto glass, and cured under 405-nm ultraviolet (UV) light in a UV oven for 10 min. All cured dielectric films were detached from the glass substrates. Combined dielectrics were prepared either by curing one dielectric directly onto the counter dielectric substrate or by laminating two freestanding dielectric films. Al tape was attached to the edge of the base dielectric layer to serve as the electrode.

### Energy band measurements

The VBM and Fermi level of each dielectric material were measured using an UPS system integrated with x-ray photoelectron spectroscopy (Thermo Fisher Scientific, NEXSA G2). A He I (21.2 eV) excitation source was used to generate photoelectrons, and the resulting electron energy distribution was analyzed to determine the SE cutoff and VBM. By accurately referencing the SE cutoff position, the Fermi level of each material was precisely determined. The UPS system was calibrated using known metallic (Au) and semiconductor (SiN*_x_*) references to ensure the accuracy of the Fermi level and VBM measurements. The bandgap energy of the materials was measured using REELS in another x-ray photoelectron spectroscopy instrument (Thermo Fisher Scientific, ESCALAB 250 Xi). Calibration for the REELS measurements was performed using a known reference material (SiN*_x_*) to ensure accurate bandgap determination. Data were analyzed by fitting the energy loss spectrum and extracting the energy threshold, which was used to calculate the bandgap value.

### Mechanical stimulation and electrical measurement

An XYZ linear actuator (Chongqing UMot Technology, China; max. speed: 200 mm/s; max. acceleration: 3000 mm/s^2^; accuracy: 50 μm) was used for mechanical stimulation, ensuring precise control over position and dynamics. The stimulating object was three-dimensionally printed and attached to the linear actuator, with a 10-mm-diameter rod covered with nitrile used for electrical signal transmission tests, and a 150- by 40-mm plate coated with Al and nitrile used for the charging process. For time-stamping sensor application tests, a 47- by 47-mm nitrile-coated plate was additionally printed and used as the stimulating object. The charging process frequency was set to 2.5 Hz, and tests were conducted for 5 min. The output electrical signals were measured in their pristine state and after the charging process with Al and nitrile at various *SP*s ranging from 0 to 132 mm from the electrode at a stimulating velocity of 145 mm/s. Oscilloscopes (Tektronix DPO2004B and RIGOL DHO5108), a digital multimeter (DMM; Keysight, 34401A, 1 kS/s), and a data acquisition system (DAQ; National Instruments, USB-6351; sampling rate: 1.25 MS/s; accuracy: <50 μV) were used to measure the output electrical signals of the test devices with an external load of 10 MΩ. The electrical measurements were conducted within a Faraday cage to minimize the electromagnetic interference. The transistor tests were performed using a probe station system with a direct current source measure unit (Keithley, 4200-SCS).

### Fabrication of time-stamping tactile sensors

The time-stamping tactile sensor was fabricated via a sequential layer-stacking process. A glass substrate was first cleaned with acetone and dried at room temperature. Adhesive copper tape was attached to the substrate at a spacing of 41 mm as bottom electrodes. The top electrodes were subsequently aligned perpendicularly to the top ones and attached using the copper tape. Before attaching the top electrodes, however, they were electrically isolated from the bottom by placing squares of insulating cellulose tape onto the bottom electrodes at the points where the two electrodes would intersect. A base dielectric layer (glass) was encapsulated over the electrode array, followed by the deposition and curing of PDMS as the counter dielectric layer. The resulting multilayer structure formed a grid-type sensor array composed of dielectric heterojunctions, enabling the formation of MSPC channels.

## Supplementary Materials

### This PDF file includes:

Figs. S1 to S37 Notes S1 to S5 Table S1 References
