# Supplementary material for: A time-stamping tactile sensor enabled by pseudoconductive interface design at dielectric heterojunctions
Source: Sci Adv. 2026 Apr 22;12(17):eaec9793. doi: 10.1126/sciadv.aec9793 (PMC13101871; doi:10.1126/sciadv.aec9793)
Supplement: Supplementary file 1 — Figs. S1 to S37 Notes S1 to S5 Table S1 References [file sciadv.aec9793_sm.pdf]

Science Advances
AAAS

# Supplementary Materials for

## A time-stamping tactile sensor enabled by pseudoconductive interface design at dielectric heterojunctions

Byungseok Seo *et al.*

Corresponding author: Xinqi Chen, xchen@northwestern.edu; Wonjoon Choi, wojchoi@korea.ac.kr

*Sci. Adv.* **12**, eaec9793 (2026)
DOI: 10.1126/sciadv.aec9793

**This PDF file includes:**

Figs. S1 to S37
Notes S1 to S5
Table S1
References

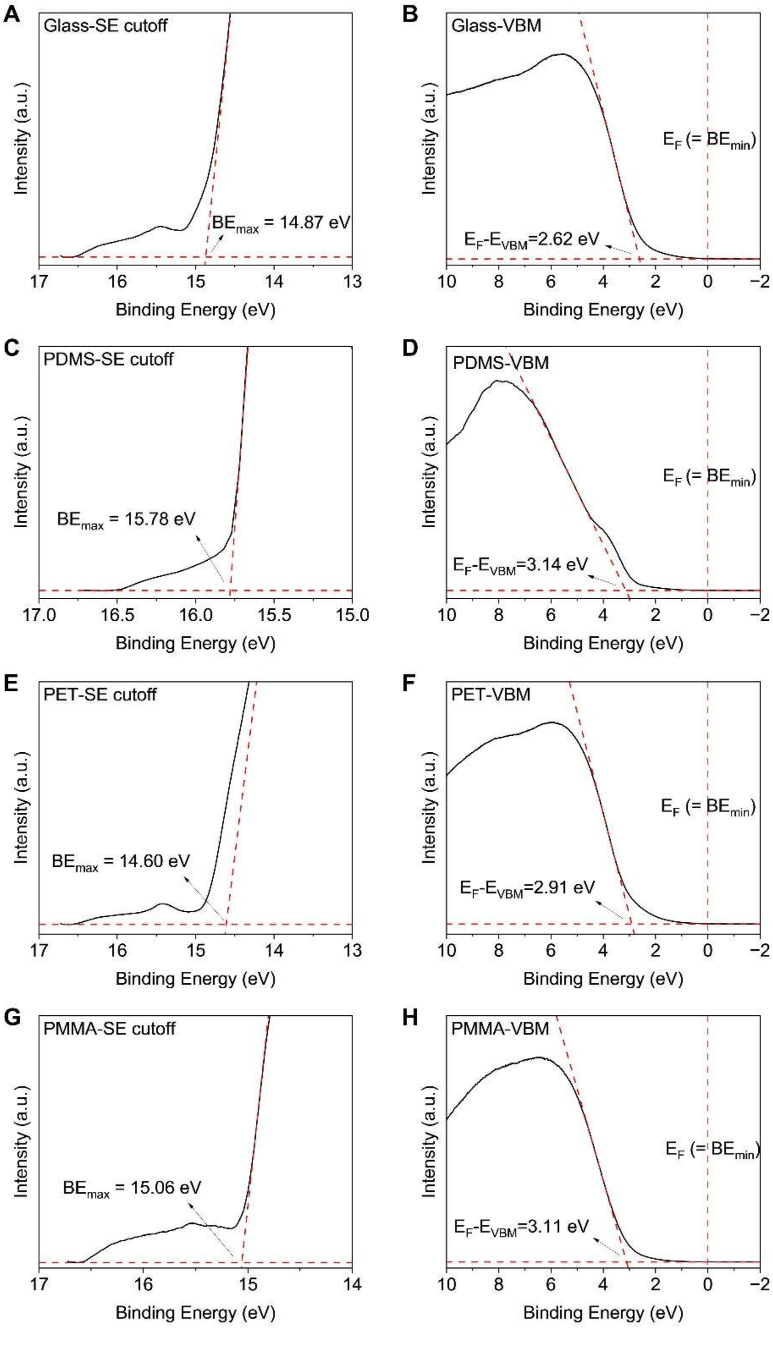


**Fig. S1**. **Ultraviolet photoelectron spectroscopy (UPS) measurements for determining the valence band maximum (VBM) and Fermi level of individual dielectrics.** Secondary electron (SE) cutoff and VBM analysis of **(A-B)** Glass, **(C-D)** polydimethylsiloxane (PDMS), **(E-F)** polyethylene terephthalate (PET), **(G-H)** polymethyl methacrylate (PMMA) specimens at their pristine state.

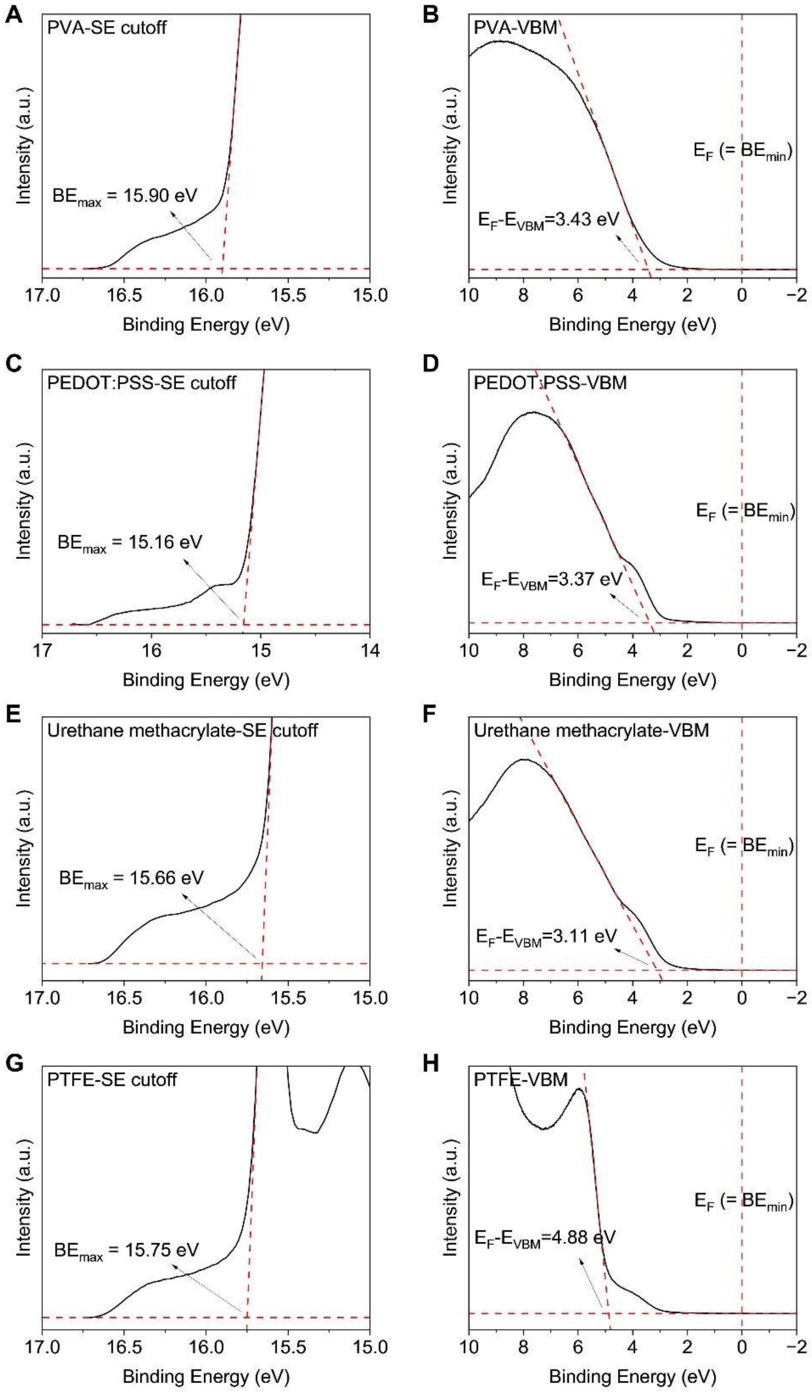


**Fig. S2**. **UPS measurements for determining the VBM and Fermi level of individual dielectrics.** SE cutoff and VBM analysis of **(A-B)** polyvinyl alcohol (PVA), **(C-D)** poly (3,4-ethylenedioxythiophene) polystyrene sulfonate (PEDOT:PSS), **(E-F)** urethane methacrylate (UMA), **(G-H)** polytetrafluoroethylene (PTFE) specimens at their pristine state.

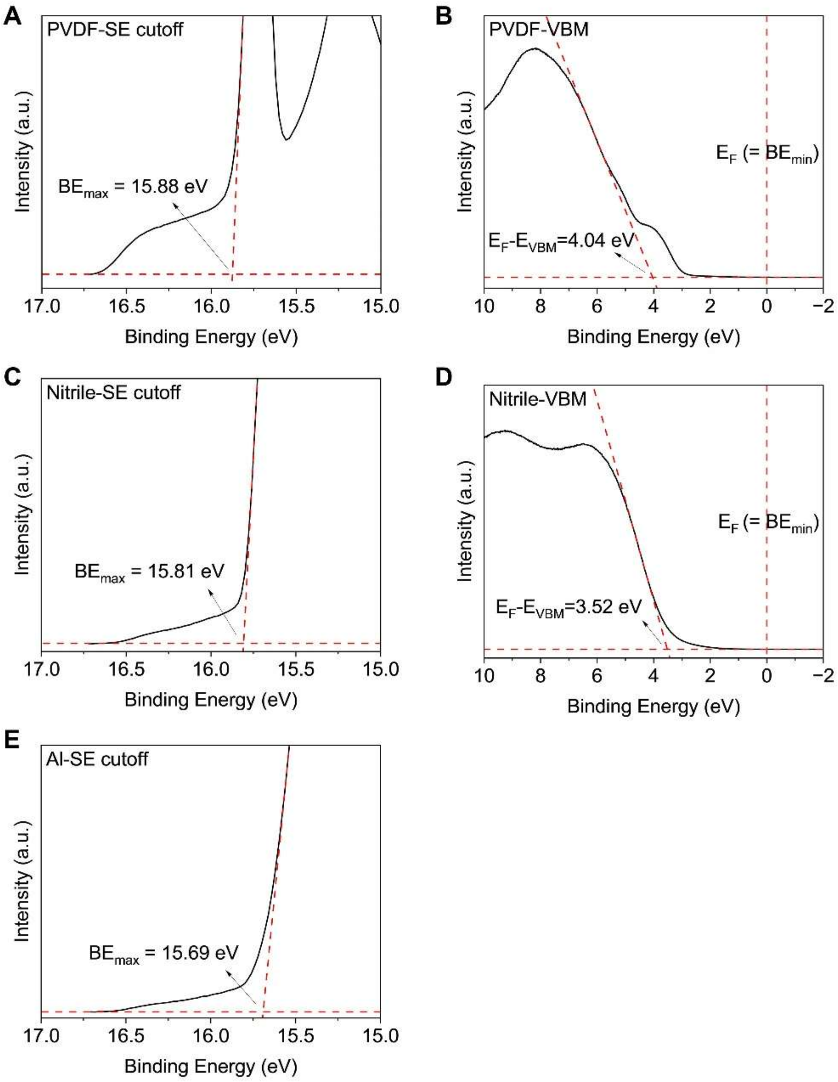


**Fig. S3**. **UPS measurements for determining the VBM and Fermi level of individual dielectrics.** SE cutoff and VBM analysis of **(A-B)** polyvinylidene fluoride (PVDF), **(C-D)** nitrile, **(E)** aluminum (Al) specimens at their pristine state.

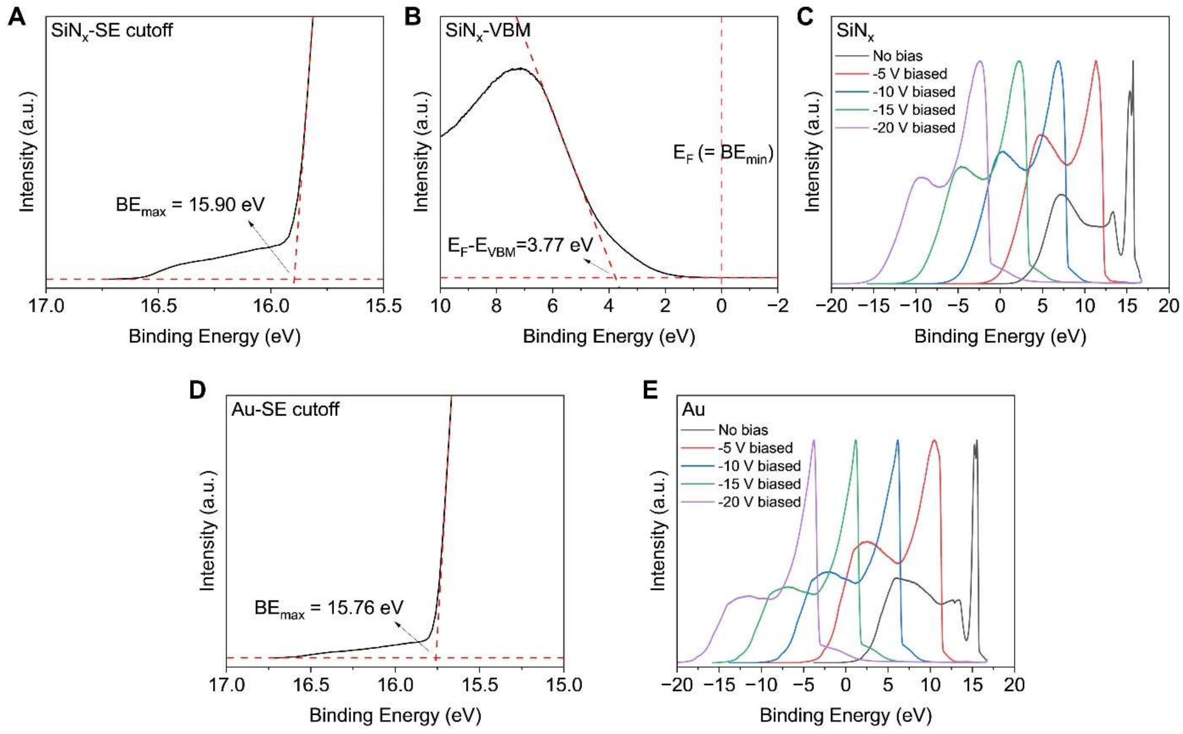


**Fig. S4**. **UPS measurements for determining the VBM and Fermi level of individual dielectrics.** SE cutoff, VBM analysis, and various biased measurements of **(A-C)** silicon nitride ($SiN_x$) and **(D-E)** gold (Au) specimens in their pristine state. These measurements are used as references for calibration to ensure the accuracy of the UPS measurements.

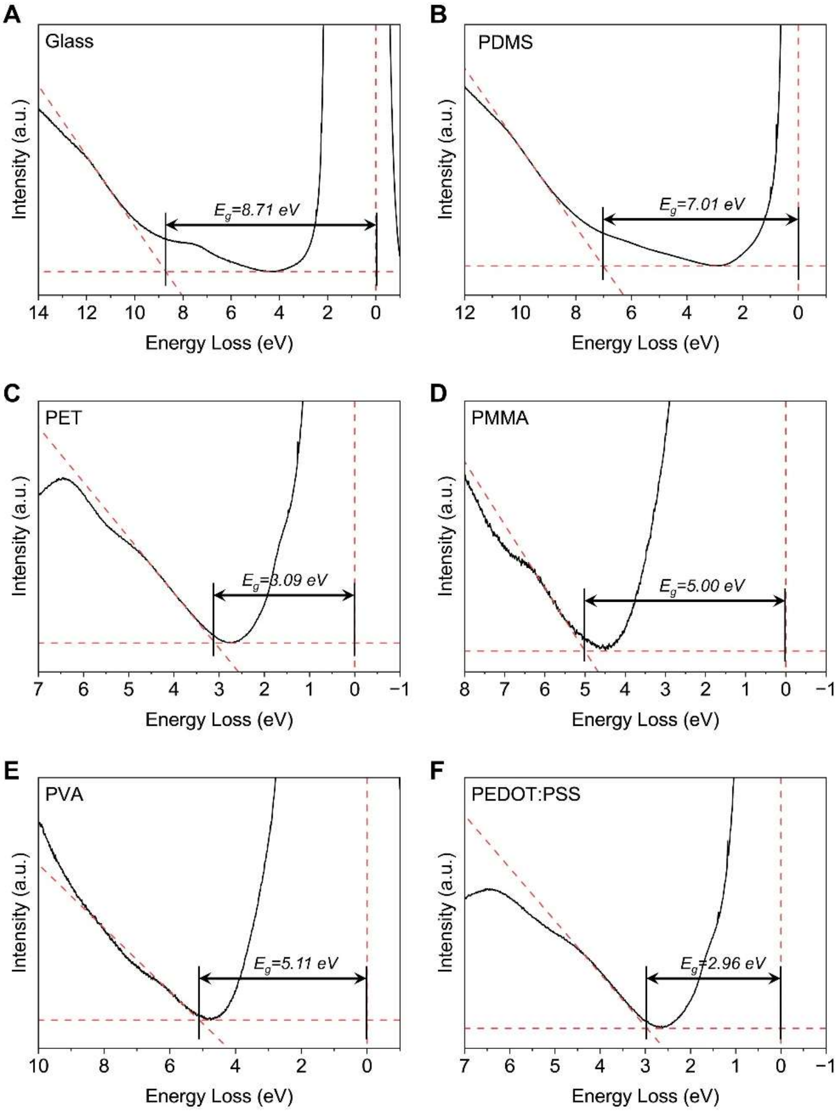


**Fig. S5**. **Reflected electron energy loss spectroscopy (REELS) spectra for bandgap measurement of individual dielectrics.** Bandgap analysis for **(A)** Glass, **(B)** PDMS, **(C)** PET, **(D)** PMMA, **(E)** PVA, and **(F)** PEDOT:PSS, determined to be 8.71 eV, 7.01 eV, 3.09 eV, 5.00 eV, 5.11 eV, and 2.96 eV, respectively, in their pristine state.

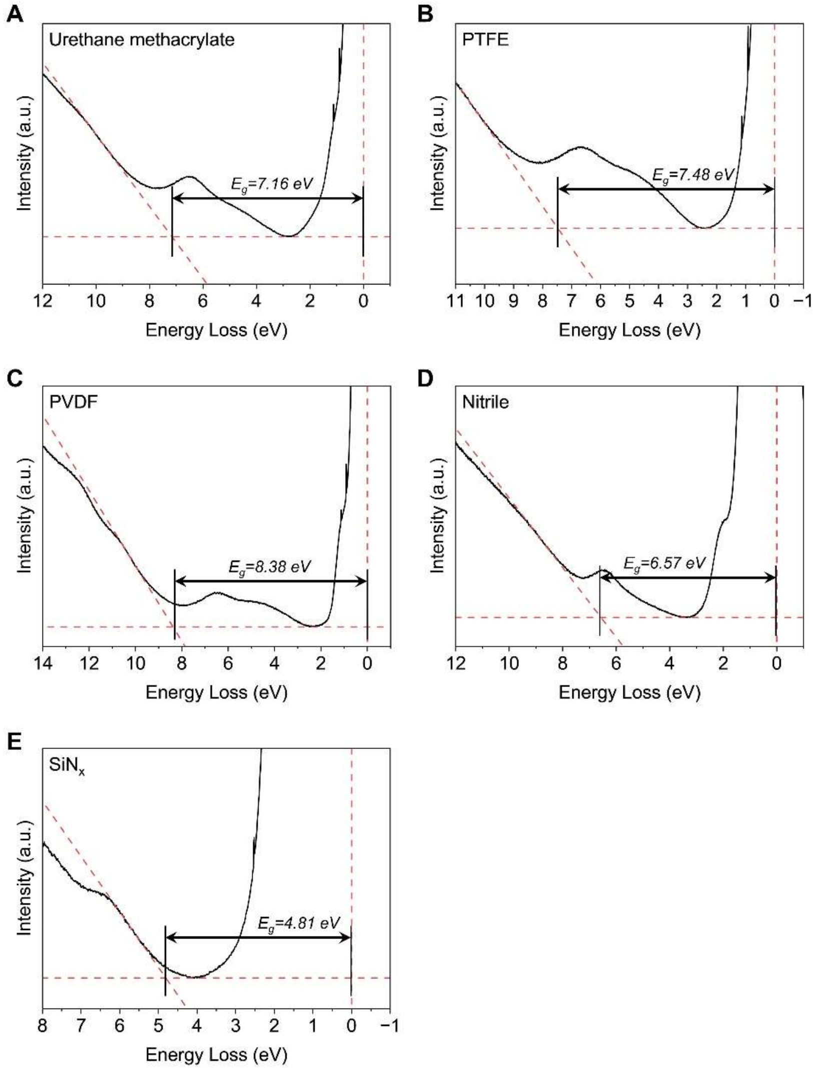


**Fig. S6**. **REELS spectra for bandgap measurement of individual dielectrics.** Bandgap analysis for **(A)** UMA, **(B)** PTFE, **(C)** PVDF, **(D)** nitrile, and **(E)** $SiN_x$, determined to be 7.16 eV, 7.48 eV, 8.38 eV, 6.57 eV, and 4.81 eV, respectively, in their pristine state. $SiN_x$ was used as a reference material for the calibration of REELS measurements.

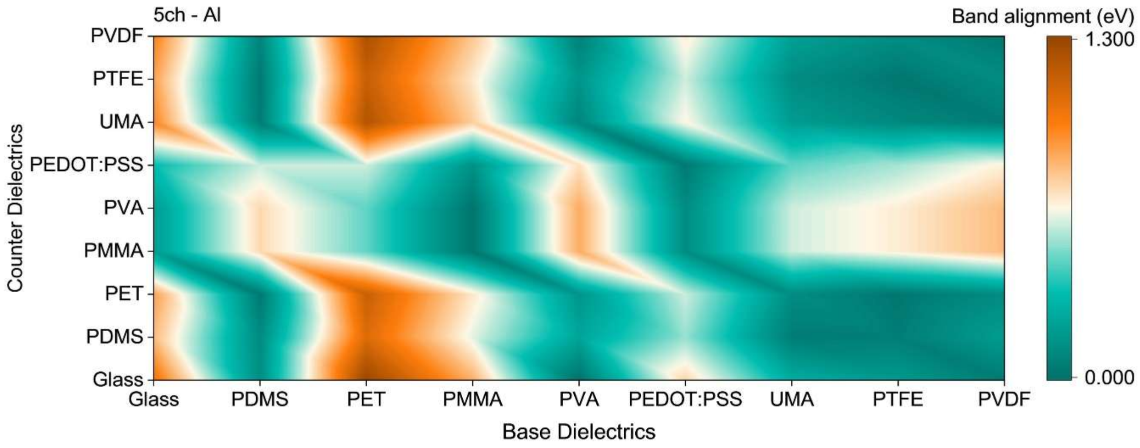


**Fig. S7**. **Evaluation of band alignment amount after charging process with stimulating objects of Al at a frequency of 2.5 Hz for 5 minutes.**

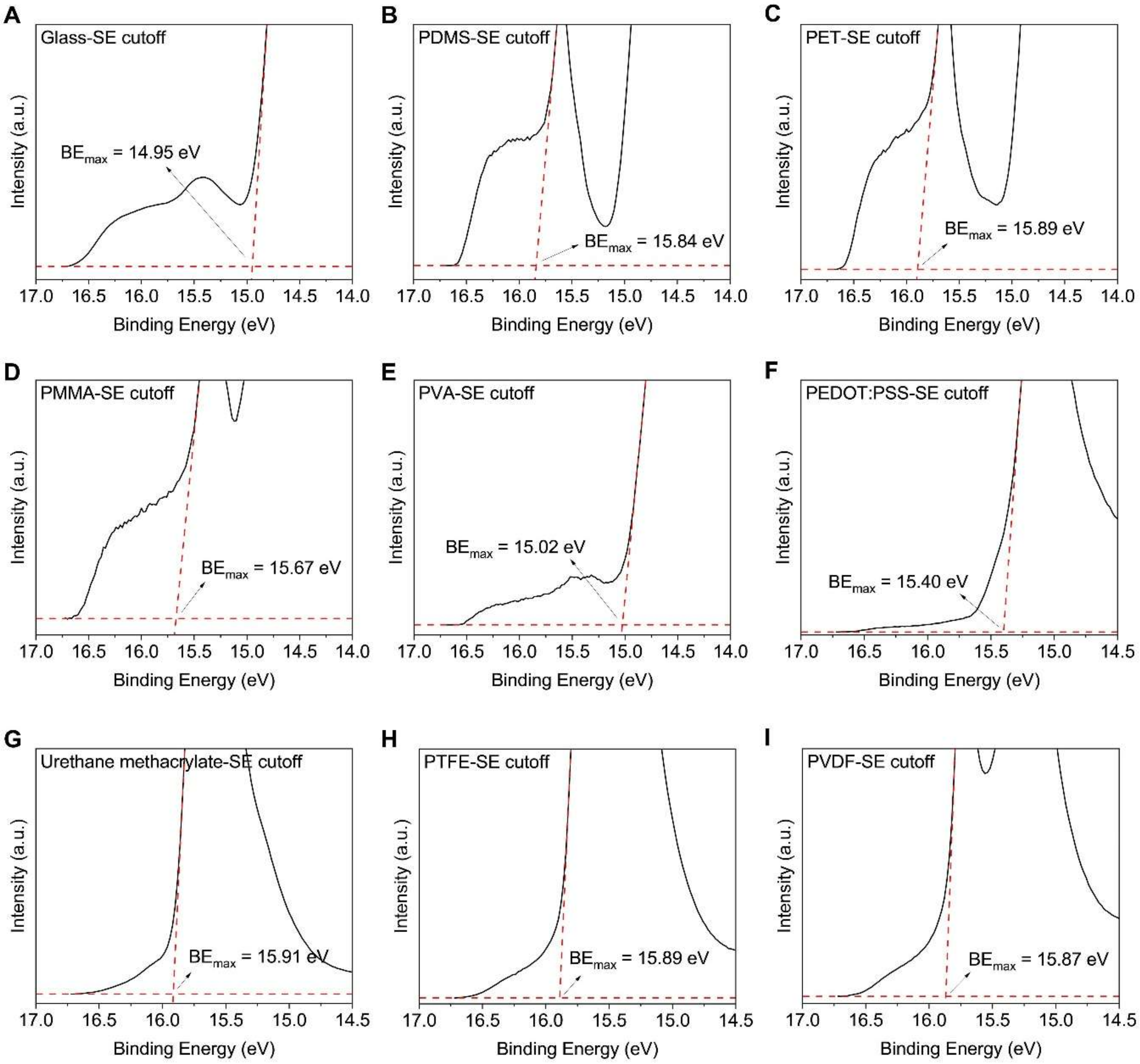


**Fig. S8**. **UPS measurements for determining the Fermi level of combined dielectrics after charging process with nitrile for 5 minutes.** SE cutoff analysis of **(A)** Glass, **(B)** PDMS, **(C)** PET, **(D)** PMMA, **(E)** PVA, **(F)** PEDOT:PSS, **(G)** UMA, **(H)** PTFE, and **(I)** PVDF specimens after charging process.

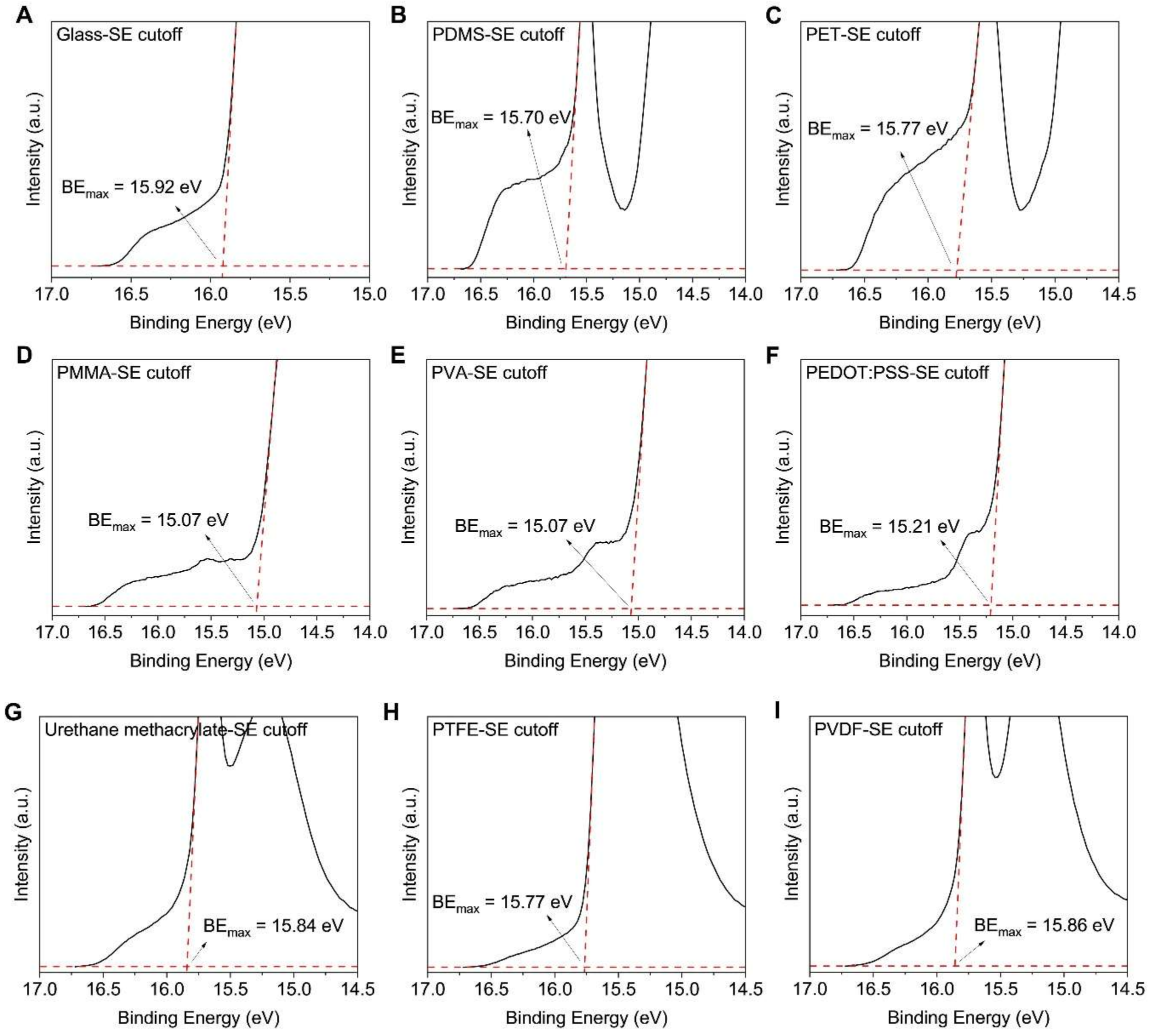


**Fig. S9**. **UPS measurements for determining the Fermi level of combined dielectrics after charging process with Al for 5 minutes.** SE cutoff analysis of **(A)** Glass, **(B)** PDMS, **(C)** PET, **(D)** PMMA, **(E)** PVA, **(F)** PEDOT:PSS, **(G)** UMA, **(H)** PTFE, and **(I)** PVDF specimens after charging process.

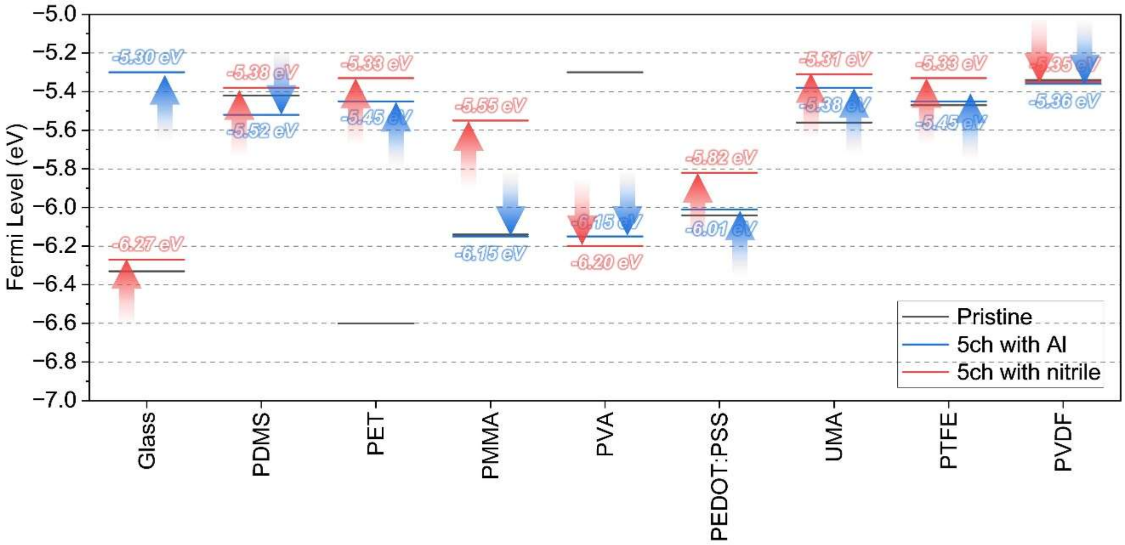


**Fig. S10**. **Evaluation of the Fermi level shift following the charging process using stimulating objects coated with nitrile and Al, performed at a frequency of 2.5 Hz for a duration of 5 minutes.**

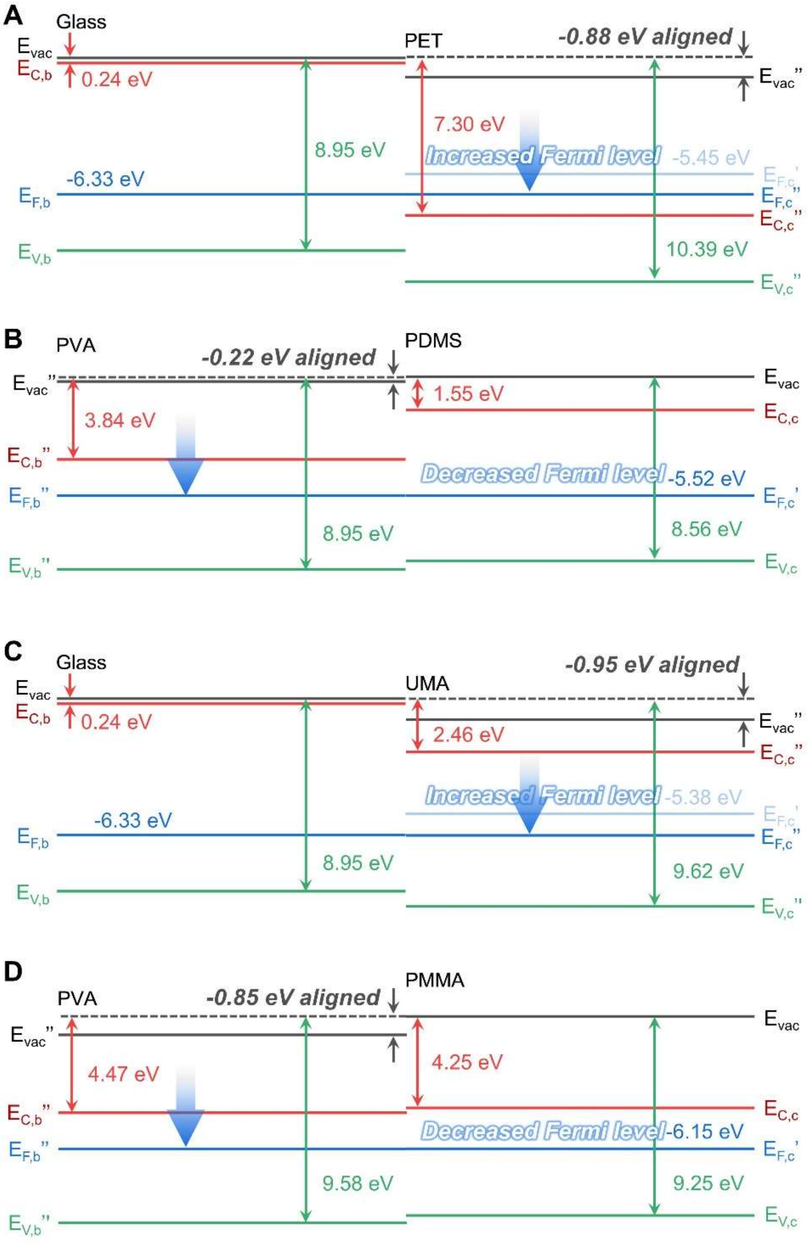


**Fig. S11**. **Examples of energy band restructuring in combined dielectrics after the charging process with an Al stimulating object at a frequency of 2.5 Hz for 5 minutes.** Energy band diagrams illustrating assured MSPC channel formation with **(A)** increased and **(B)** decreased Fermi levels in Glass/PET and PVA/PDMS combinations, respectively. Energy band diagrams of potential MSPC channel formation, achieved after the charging process with **(C)** increased and **(D)** decreased Fermi levels in Glass/UMA and PVA/PMMA combinations.

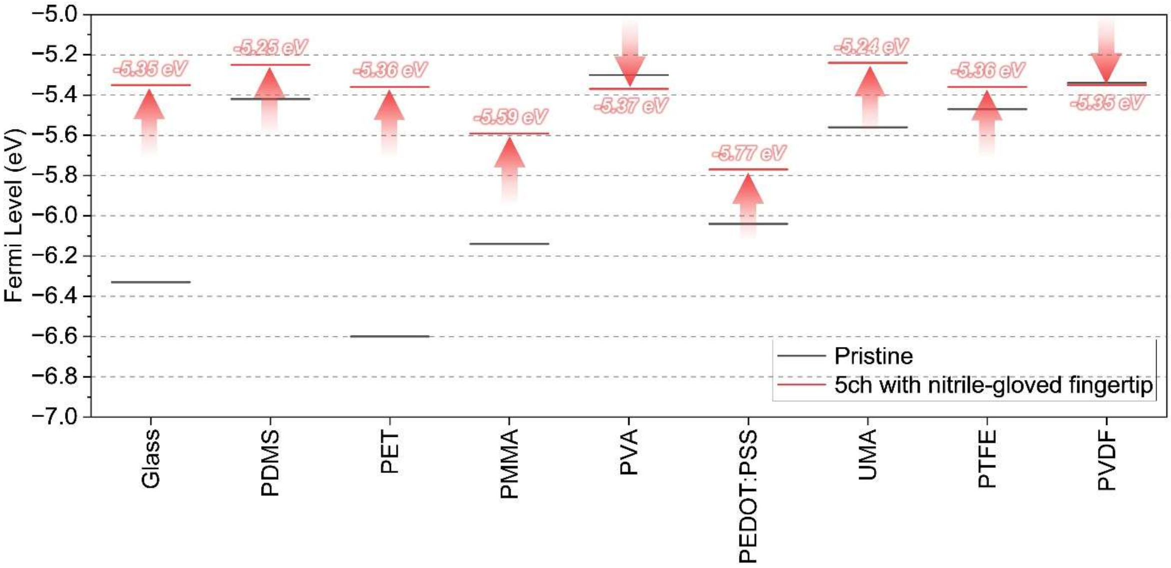


**Fig. S12**. **Assessment of the Fermi level shift after the charging process using a nitrile-gloved fingertip touch at a frequency of 2.5 Hz for a duration of 5 minutes.**

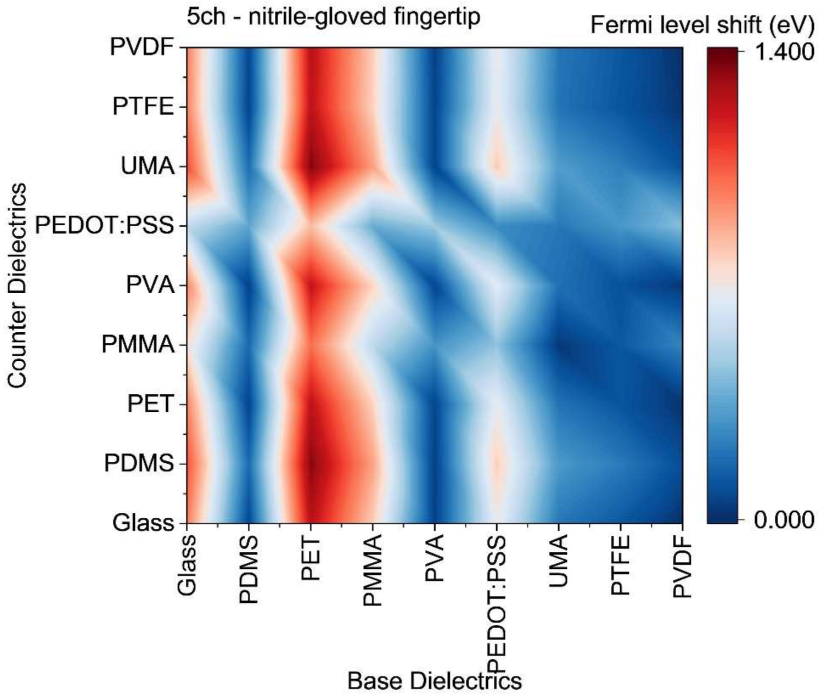


**Fig. S13**. **Evaluation of Fermi level shift after a 5-minute tapping process with a nitrile-gloved fingertip.** This assessment was conducted to observe the restructuring of the energy band in combined dielectrics induced by mechanical stimulation.

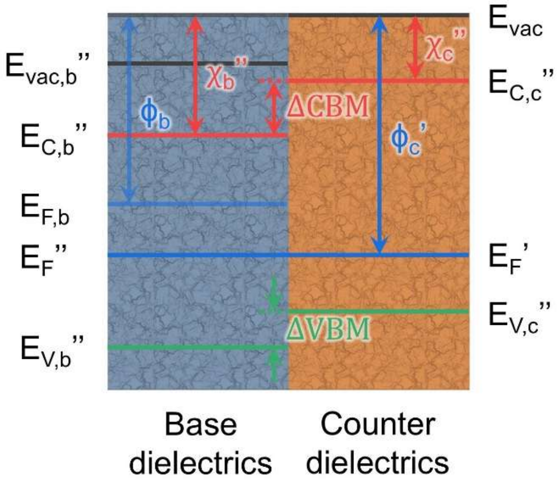


**Fig. S14**. **Energy band diagrams illustrating parameter designations for establishing the MSPC favorability coefficient (MFC) and MSPC favorability index (MFI).** E, E', and E'' denote the energy levels in the pristine state, immediately after the charging process, and after band alignment, respectively. $\phi$ and $\phi$' indicate the work function in the pristine state and immediately after the charging process, respectively, while $\chi$'' represents the charge affinity after the charging process. Subscripts b, c, and o refer the base dielectrics, counter dielectrics, and stimulating object, respectively.

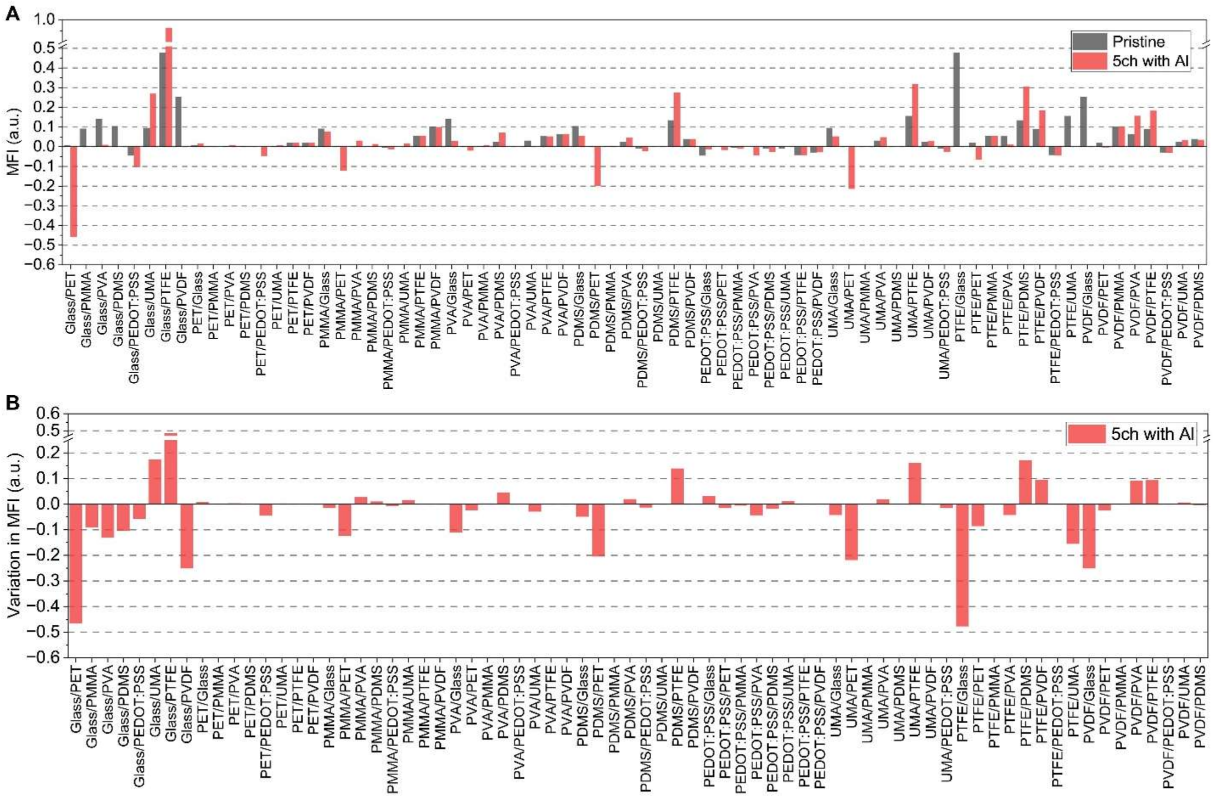


**Fig. S15**. **Evaluation of the MFI for combined dielectrics. (A)** MFI values of combined dielectrics consisting of base and counter dielectrics in their pristine state and after an 5-minute charging process with Al, considering variations in the conduction band minimum (CBM), VBM, charge affinity ($\chi$), and band tilting propensity ($P_{BT}$). **(B)** Comparison of MFI changes after the charging process with Al relative to the pristine values.

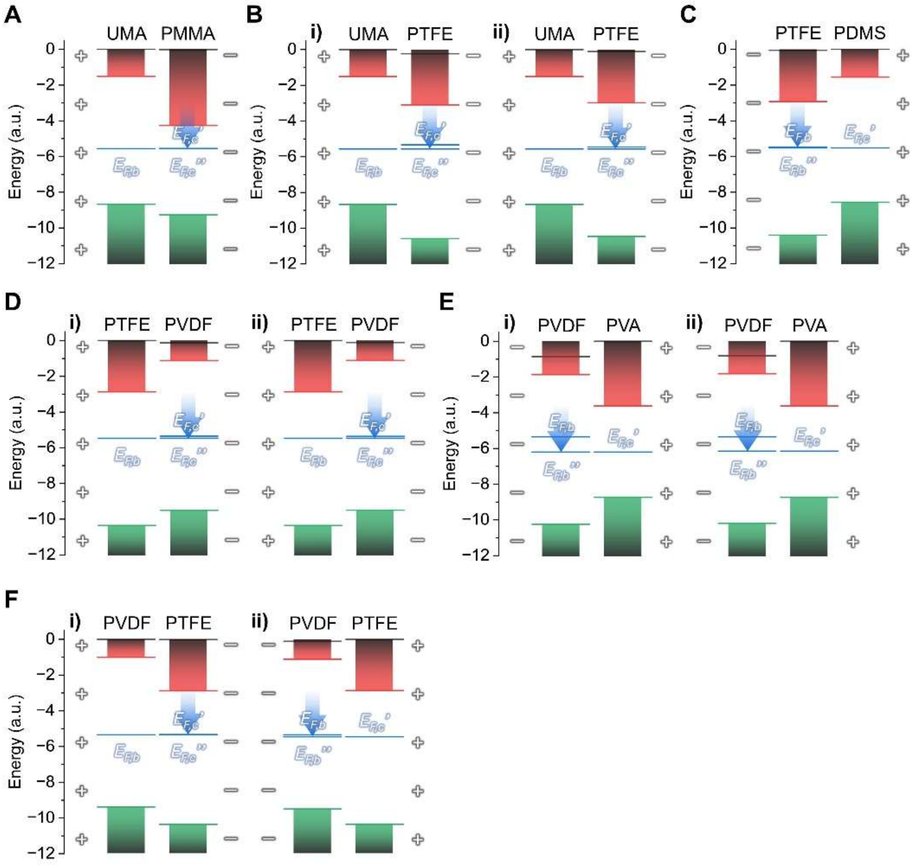


**Fig. S16**. **Energy band alignment of combined dielectrics with high MFI values after the charging process. (A)** UMA/PMMA combination after 5ch with nitrile (MFI~0.10). **(B)** UMA/PTFE combination after 5ch with (i) nitrile (MFI~0.35) and (ii) Al (MFI~0.32). **(C)** PTFE/PDMS combination after 5ch with Al (MFI~0.31). **(D)** PTFE/PVDF combination after 5ch with (i) nitrile (MFI~0.18) and (ii) Al (MFI~0.18). **(E)** PVDF/PVA combination after 5ch with (i) nitrile (MFI~0.16) and (ii) Al (MFI~0.16). **(F)** PVDF/PTFE combination after 5ch with (i) nitrile (MFI~0.21) and (ii) Al (MFI~0.18).

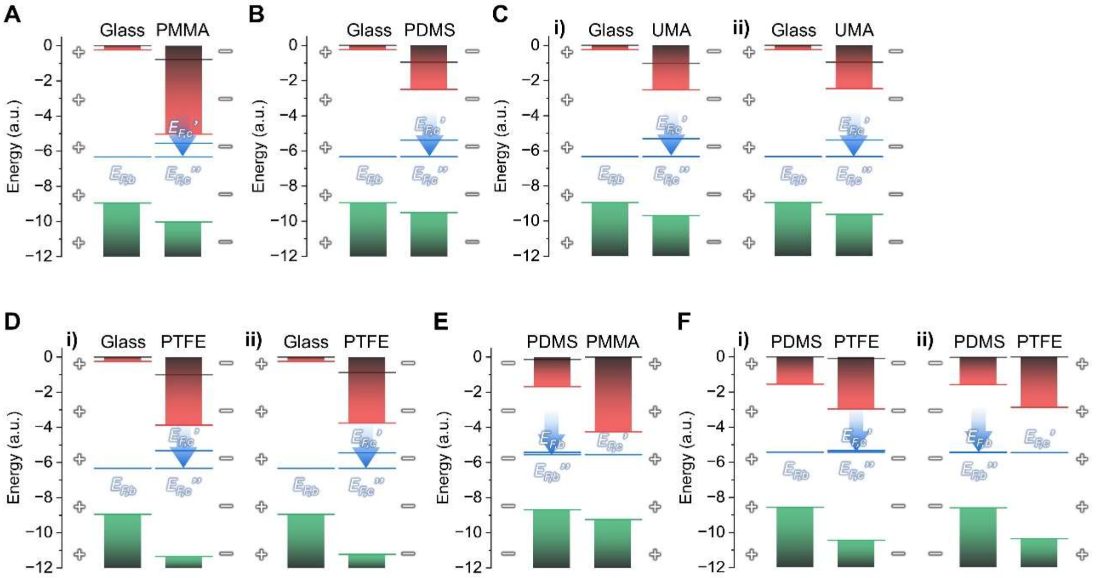

**Fig. S17**. **Energy band alignment of combined dielectrics with high MFI values after the charging process. (A)** Glass/PMMA (MFI~0.31) and **(B)** Glass/PDMS (MFI~0.23) combinations after 5ch with nitrile. **(C)** Glass/UMA combination after 5ch with (i) nitrile (MFI~0.30) and (ii) Al (MFI~0.27). **(D)** Glass/PTFE combination after 5ch with (i) nitrile (MFI~1.00) and (ii) Al (MFI~0.96). **(E)** PDMS/PMMA combination after 5ch with nitrile (MFI~0.09). **(F)** PDMS/PTFE combination after 5ch with (i) nitrile (MFI~0.30) and (ii) Al (MFI~0.27).

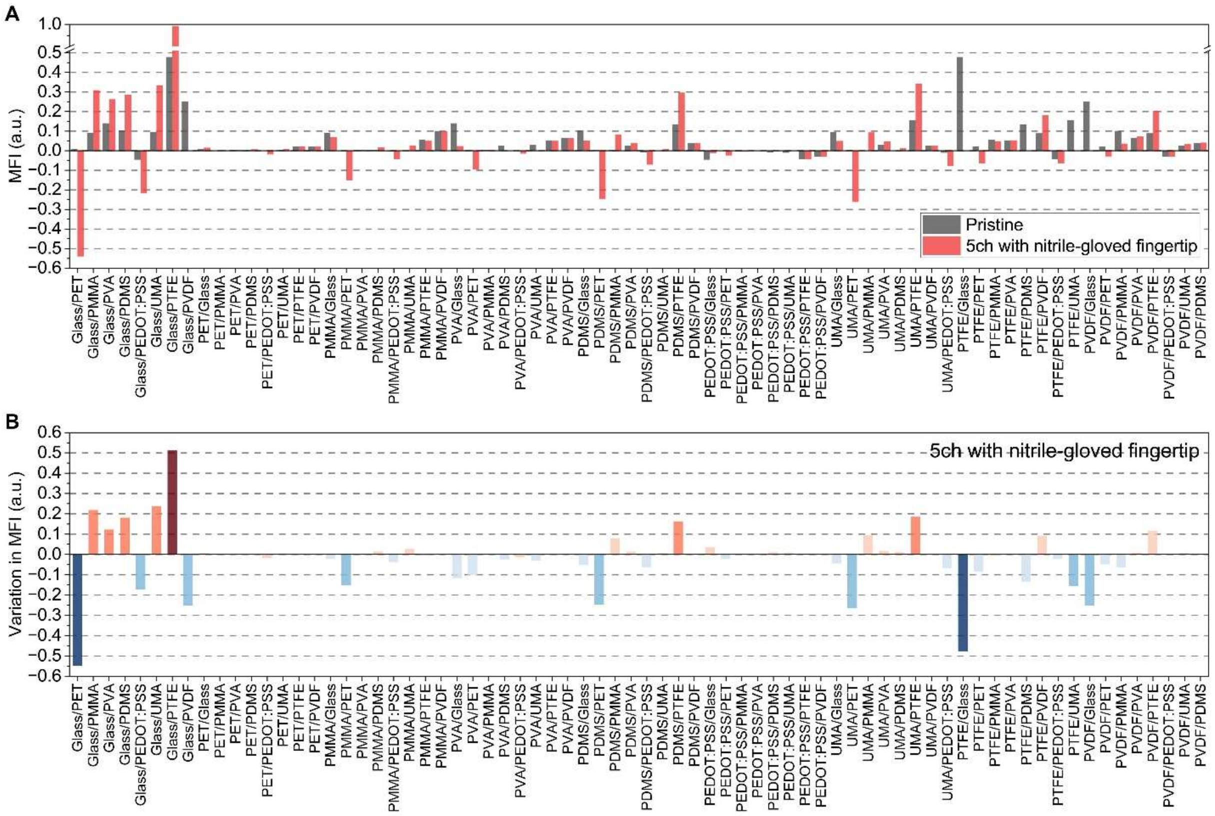


**Fig. S18**. **Evaluation of the MFI for combined dielectrics.** **(A)** MFI values of combined dielectrics consisting of base and counter dielectrics in their pristine state and after an 5-minute charging process with a nitrile-gloved fingertip, considering variations in the conduction band minimum (CBM), VBM, charge affinity ($\chi$), and band tilting propensity ($P_{BT}$). **(B)** Comparison of MFI changes after the charging process with a nitrile-gloved fingertip relative to the pristine values.

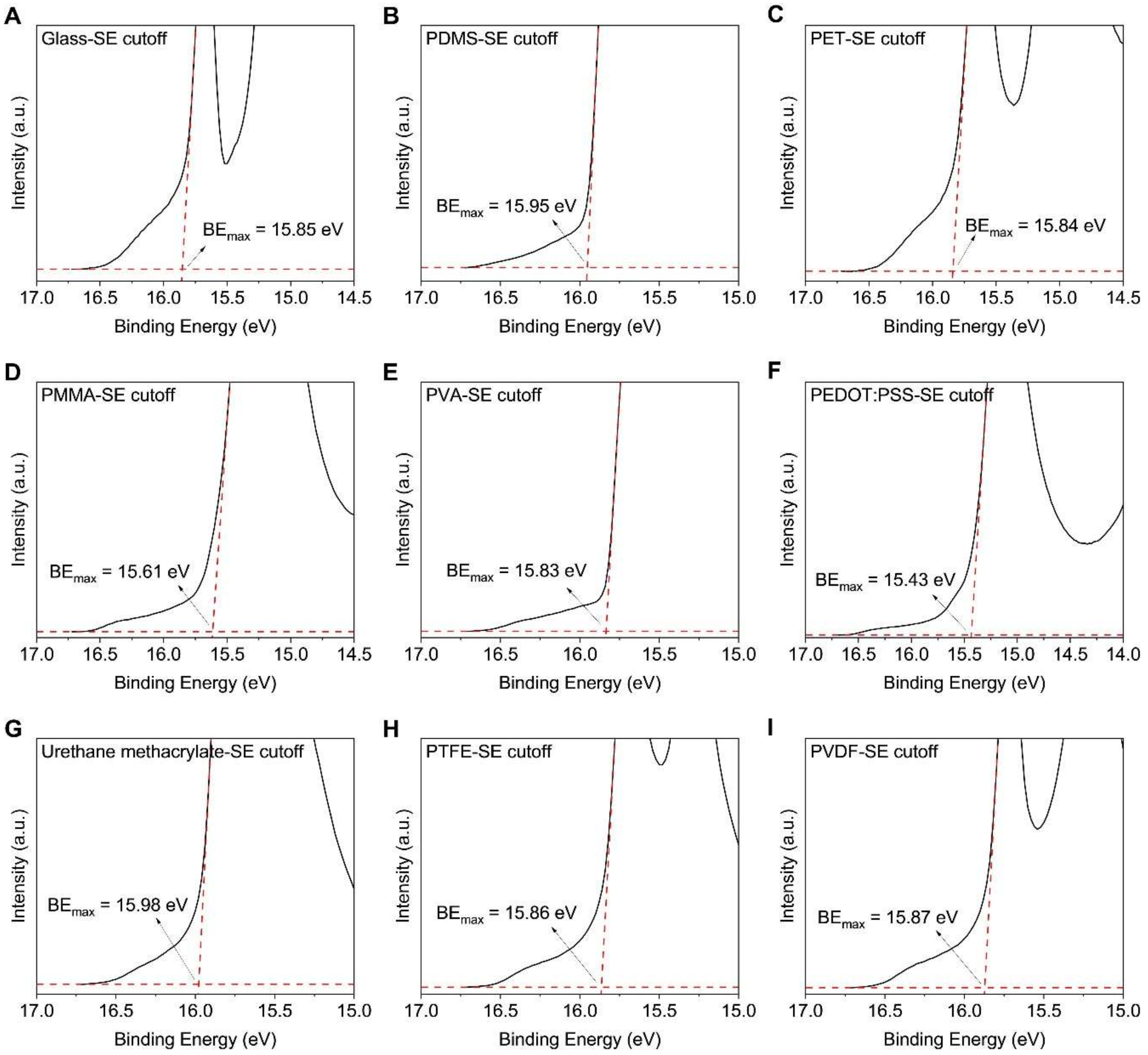


**Fig. S19**. **UPS measurements for determining the Fermi level of combined dielectrics after a 5-minute charging process with a nitrile-gloved fingertip.** SE cutoff analysis of **(A)** Glass, **(B)** PDMS, **(C)** PET, **(D)** PMMA, **(E)** PVA, **(F)** PEDOT:PSS, **(G)** UMA, **(H)** PTFE, and **(I)** PVDF specimens following the charging process.

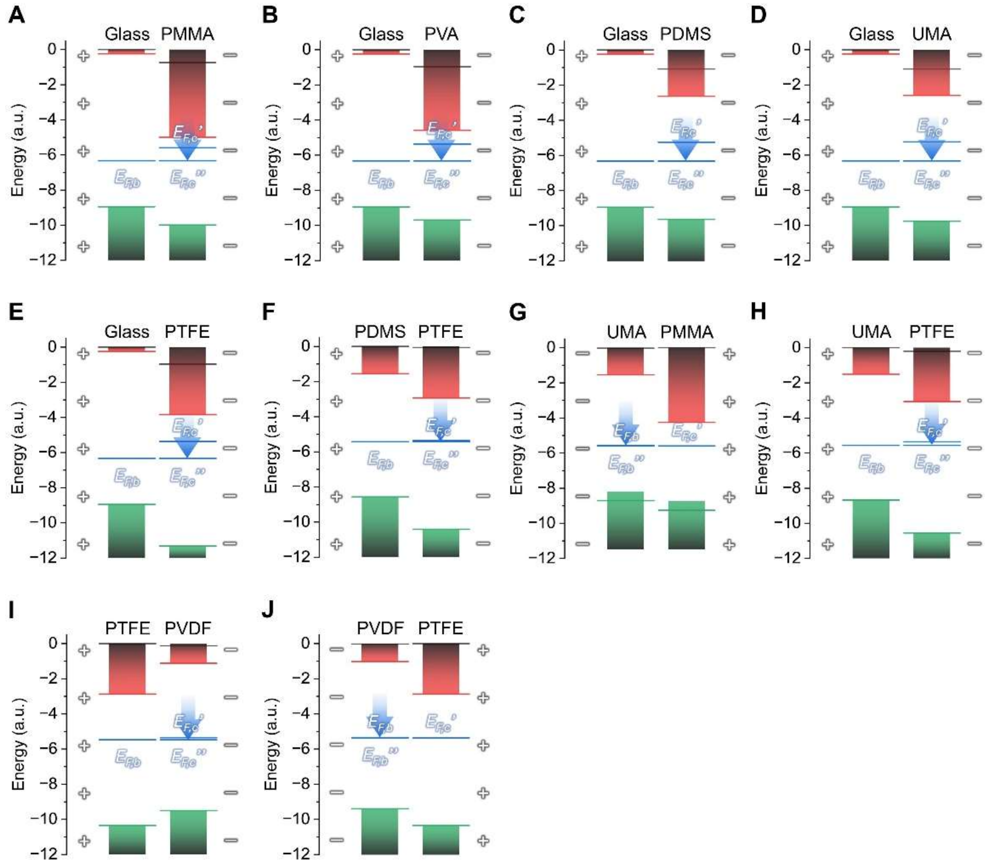


**Fig. S20**. **Energy band alignment of combined dielectrics with high MFI values after the charging process with nitrile-gloved fingertip for 5 minutes.** **(A)** Glass/PMMA (MFI~0.31), **(B)** Glass/PVA (MFI~0.26), **(C)** Glass/PDMS (MFI~0.28), **(D)** Glass/UMA (MFI~0.33), **(E)** Glass/PTFE (MFI~0.99), **(F)** PDMS/PTFE (MFI~0.30), **(G)** UMA/PMMA (MFI~0.093), **(H)** UMA/PTFE (MFI~0.34), **(I)** PTFE/PVDF (MFI~0.18), and **(J)** PVDF/PTFE (MFI~0.20) combinations.

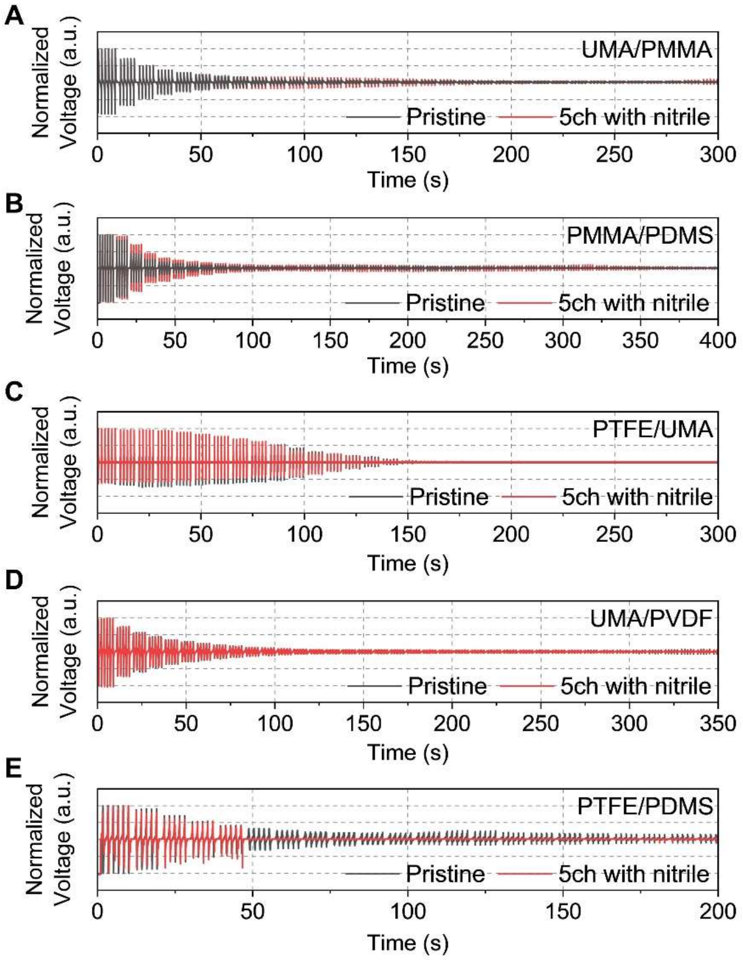


**Fig. S21**. **Normalized temporal voltage profiles of MSPC devices under increasing stimulus position (SP), generated through mechano-electric energy conversion. (A)** UMA/PMMA, **(B)** PMMA/PDMS, **(C)** PTFE/UMA, **(D)** UMA/PVDF, and **(E)** PTFE/PDMS devices.

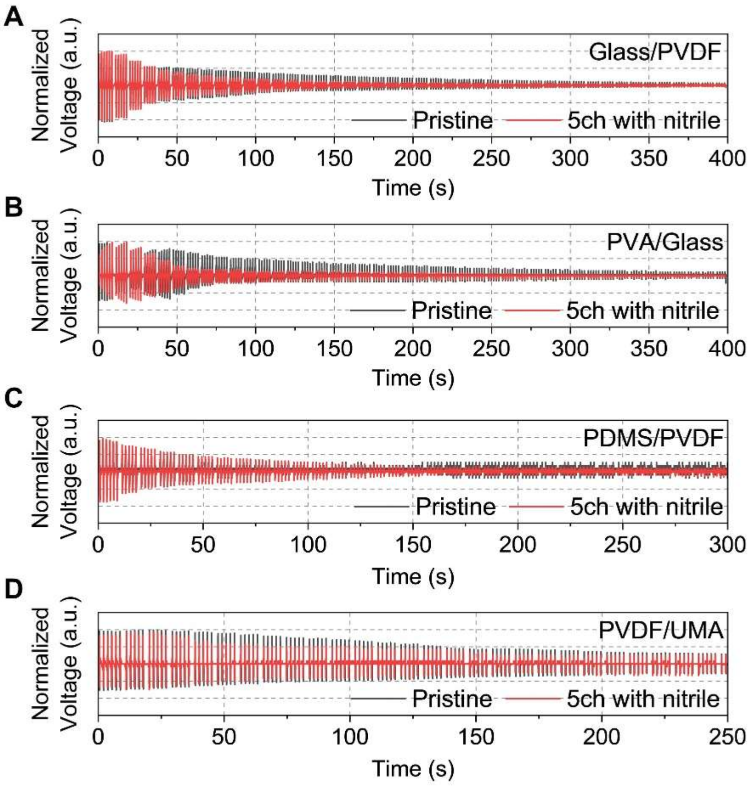


**Fig. S22. Normalized temporal voltage profiles of MSPC devices under increasing SP, generated through mechano-electric energy conversion. (A)** Glass/PVDF, **(B)** PVA/Glass, **(C)** PDMS/PVDF, and (d) PVDF/UMA devices.

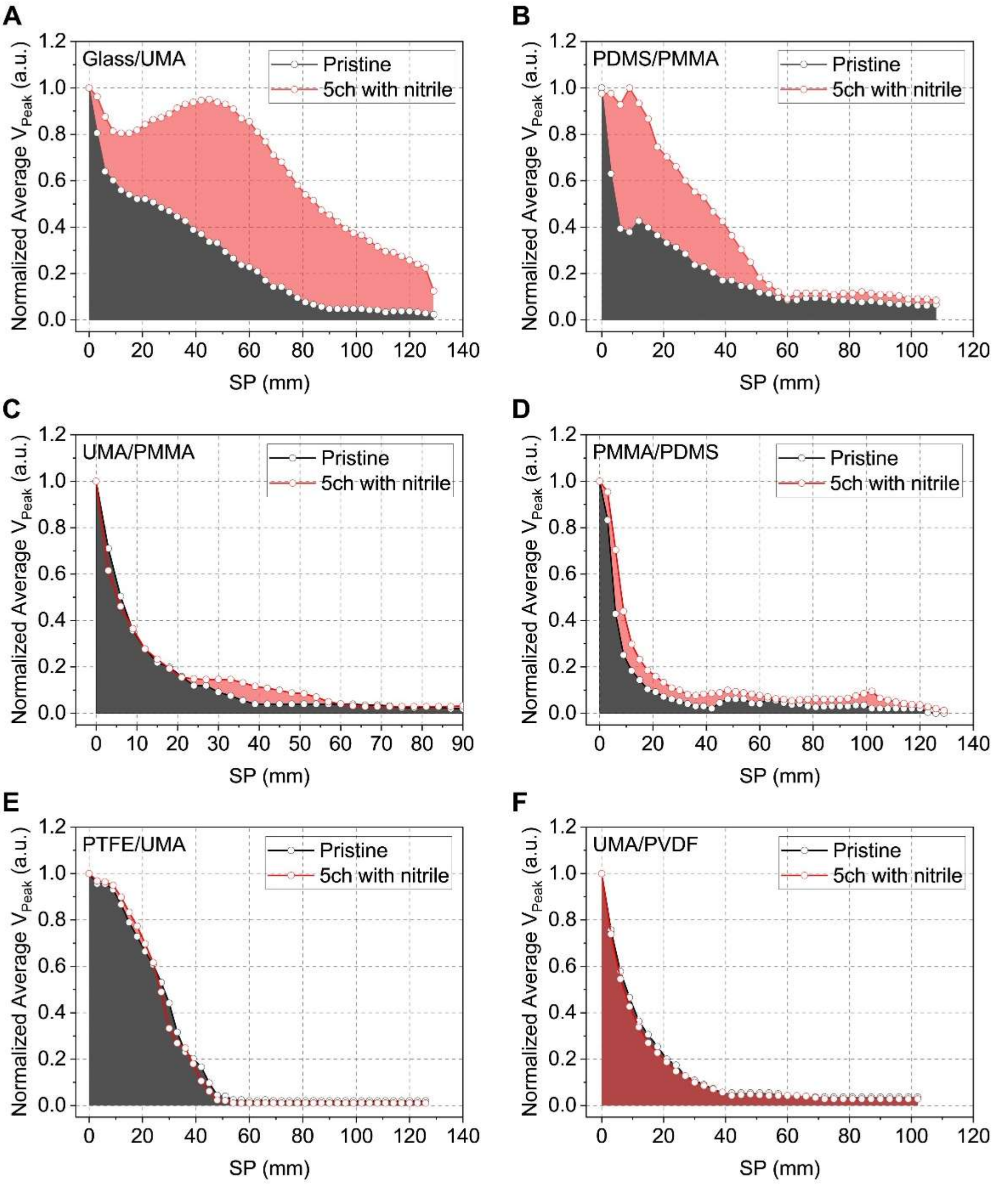


**Fig. S23**. **Normalized average voltage peak profiles of MSPC devices as a function of SP, measured before and after an 5-minute charging process with nitrile. (A)** Glass/UMA, **(B)** PDMS/PMMA, **(C)** UMA/PMMA, **(D)** PMMA/PDMS, **(E)** PTFE/UMA, and **(F)** UMA/PVDF devices.

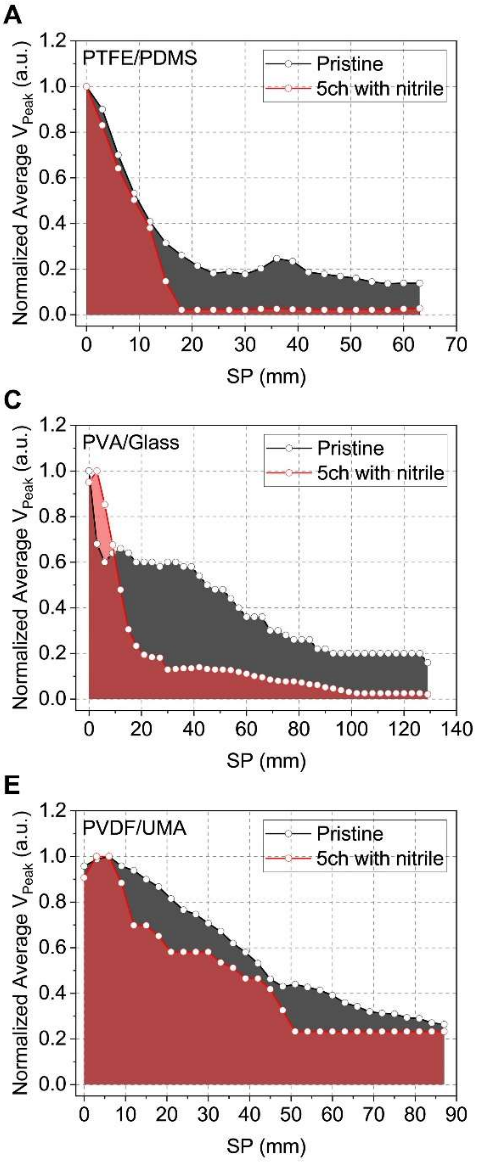


**Fig. S24**. **Normalized average voltage peak profiles of MSPC devices as a function of SP, measured before and after a 5-minute charging process with nitrile. (A)** PTFE/PDMS, **(B)** Glass/PVDF, **(C)** PVA/Glass, **(D)** PDMS/PVDF, and **(E)** PVDF/UMA devices.

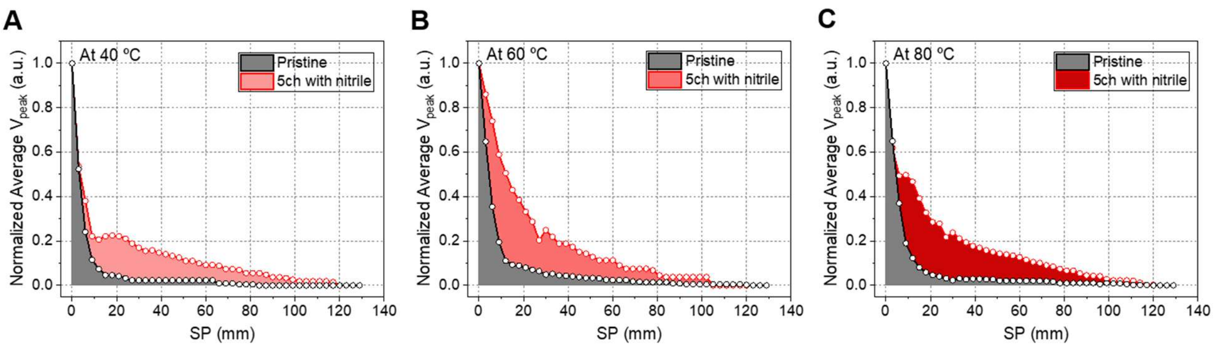


**Fig. S25. Temperature-dependent MSPC behavior.** Normalized average voltage peak profiles of glass/PDMS device as a function of SP, measured in the pristine state and after 5 min nitrile charging at different temperatures: **(A)** 40 °C, **(B)** 60 °C, and **(C)** 80 °C.

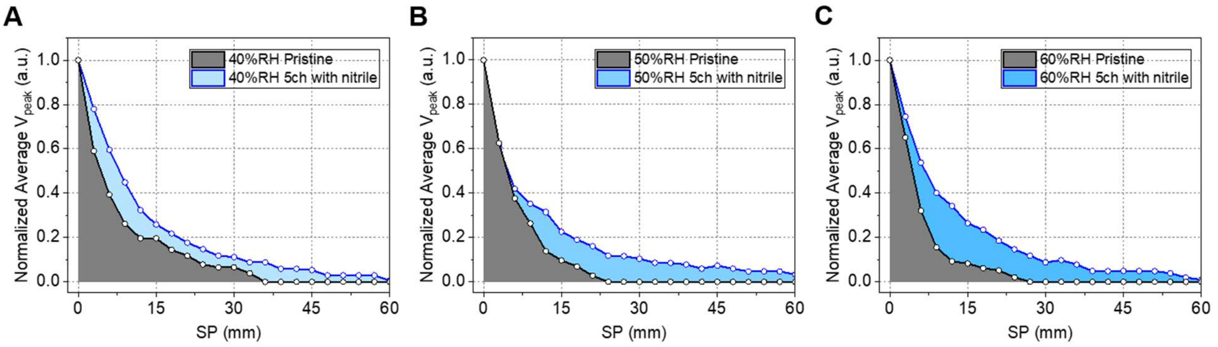


**Fig. S26. Humidity-dependent MSPC behavior.** Normalized average voltage peak profiles of glass/PDMS devices as a function of SP, measured in the pristine state and after 5 min nitrile charging under controlled relative humidities: **(A)** 40% RH, **(B)** 50% RH, and **(C)** 60% RH.

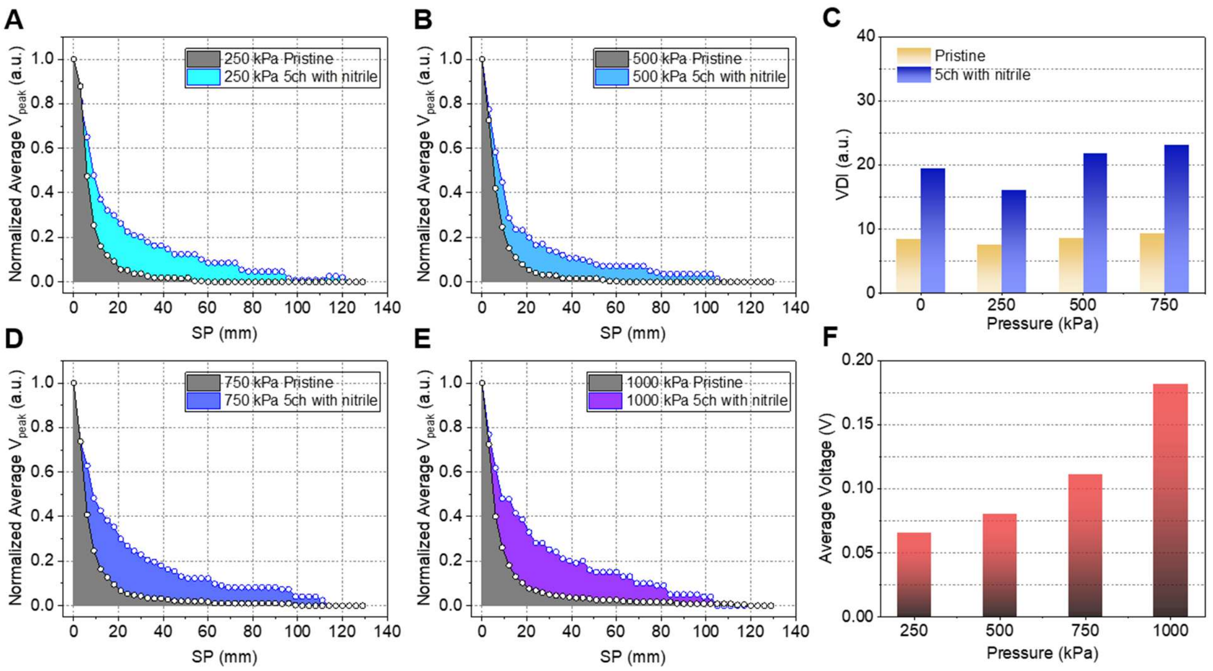


**Fig. S27. Pressure-dependent MSPC behavior and performance metrics.** Normalized average voltage peak profiles of glass/PDMS device as a function of SP measured in the pristine state and after 5 min nitrile charging under different applied pressures: **(A)** 250 kPa, **(B)** 500 kPa, **(D)** 750 kPa, and **(E)** 1000 kPa. **(C)** Comparison of VDI values before and after nitrile charging as a function of pressure. **(F)** Mean absolute output voltage as a function of applied pressure

**Note S1. Reliability of MSPC behavior under temperature, humidity, and pressure variations**

Environmental conditions such as temperature and humidity, as well as mechanical conditions such as applied stress, can affect the electrical responses of dielectric surfaces by altering surface charge dissipation pathways and the effective contact area. However, the MSPC phenomenon is activated and sustained by interfacial processes at the base–counter dielectric junction, which is spatially separated from the ambient-exposed dielectric surface. Therefore, while the pristine triboelectric output can be sensitive to external perturbations, the MSPC-mediated voltage sustainment remains robust. To verify this, we systematically evaluated MSPC behavior under controlled temperature, relative humidity, and pressure conditions, as detailed below.

i) Temperature dependence
Temperature is a frequently discussed factor in triboelectric-based devices because elevated temperature can facilitate charge dissipation through enhanced carrier activity and faster discharge pathways. Importantly, because MSPC is governed by interfacial phenomena at the base–counter dielectric junction, variations in surface discharge to the surrounding environment are expected to have only a minor effect on the MSPC response.
To examine thermal robustness, we measured the normalized voltage peak behavior as a function of SP at three temperatures: 40, 60, and 80 °C. **Fig. S25** presents the normalized voltage peak profiles obtained for each temperature condition in both the pristine state and after 5 min nitrile charging. Regardless of temperature, the pristine state exhibits rapid voltage decay, whereas after nitrile charging the voltage becomes sustained and the MSPC behavior is consistently observed. These results indicate that temperature variation does not measurably affect the interfacial MSPC mechanism.

ii) Humidity dependence
Humidity is also a major parameter that can influence triboelectric outputs because high humidity promotes charge redistribution and leakage through adsorbed water layers, thereby reducing measurable voltages. Similar to the temperature case, the pristine triboelectric output can be strongly affected by surface charge dissipation. In contrast, the MSPC response arises from interfacial processes at the base–counter dielectric junction and is expected to be retained. We therefore conducted electrical signal transmission tests at controlled relative humidities of 40% (**Fig. S26A**), 50% (**Fig. S26B**), and 60% RH (**Fig. S26C**). For each humidity condition, we examined the normalized voltage peak response as a function of SP in both the pristine state and after 5 min nitrile charging. When comparing the pristine-state responses across different relative humidity conditions, the voltage decays more rapidly at higher humidity, indicating accelerated surface charge leakage. In contrast, after 5 min nitrile charging, the voltage remains sustained and the MSPC behavior is reproduced consistently across all humidity conditions. These results confirm that the MSPC response is preserved regardless of humidity.

iii) Pressure dependence
Increasing pressure primarily enlarges the effective contact area between the stimulating object and the counter dielectric, thereby increasing the amount of charge exchange at the surface and consequently raising the absolute output voltage. However, variations in pressure do not alter the intrinsic MSPC mechanism, because MSPC is governed by interfacial processes between the base dielectric and the counter dielectric network rather than being dictated solely by the counter dielectric surface state. To verify pressure-related reliability, we performed measurements under

four pressure conditions (250, 500, 750, and 1000 kPa) and compared the normalized voltage evolution and VDI before and after 5 min nitrile charging (**Figs. S27A, B, D, E**). Across all pressure cases, the pristine state exhibits pronounced voltage decay with low VDI, whereas after nitrile charging the voltage becomes substantially sustained with markedly higher VDI (**Fig. S27C**). Notably, the VDI enhancement remains comparable across pressures, with the post-charging VDI reaching approximately double that of the pristine state, while the average absolute voltage increases monotonically with pressure **(Fig. S27F)**. Overall, these results indicate that pressure variation modulates signal magnitude but does not compromise the MSPC-enabled time-stamping behavior.

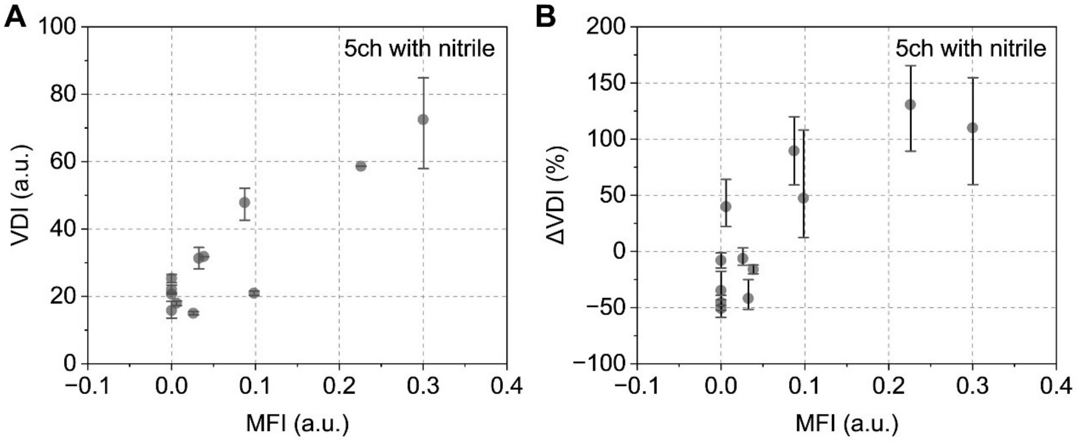


**Fig. S28**. **Relationship between (A) MFI and VDI and (B) MFI and ΔVDI after an 5-minute charging process with nitrile.** The evaluation was conducted using MSPC devices, including (1) Glass/UMA, (2) Glass/PDMS, (3) PDMS/PMMA, (4) UMA/PMMA, (5) PMMA/PDMS, (6) PTFE/UMA, (7) UMA/PVDF, (8) PTFE/PDMS, (9) Glass/PVDF, (10) PVA/Glass, (11) PDMS/PVDF, and (12) PVDF/UMA.

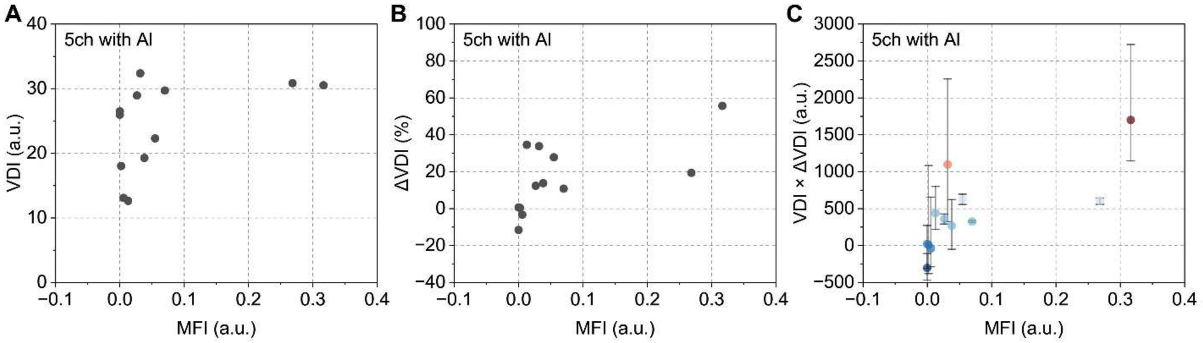


**Fig. S29**. **Relationship between (A) MFI and VDI, (B) MFI and ΔVDI, and (C) MFI and VDI × ΔVDI after an 5-minute charging process with Al.** The evaluation was conducted using MSPC devices, including Glass/UMA, Glass/PVDF, PDMS/Glass, PDMS/PVDF, PVA/PDMS, PVA/PMMA, PET/PDMS, PTFE/UMA, PMMA/PDMS, PVDF/UMA, UMA/PVDF, UMA/PTFE.

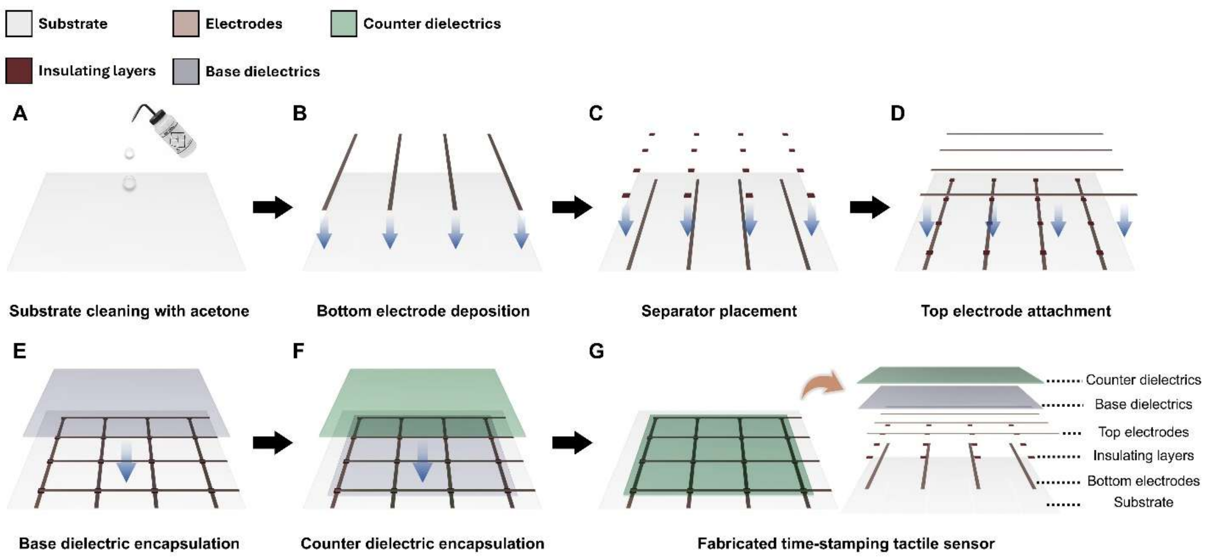


**Fig. S30**. **Fabrication process of the time-stamping tactile sensor device. (A)** Glass substrate cleaning with acetone. **(B)** Deposition of bottom copper electrodes on the substrate with predefined electrode spacing. **(C)** Placement of separators to prevent interference between the bottom and top electrodes. **(D)** Attachment of top copper electrodes. Sequential encapsulation with **(E)** base (glass) and **(F)** counter (PDMS) dielectric layers. **(G)** Final time-stamping tactile sensor and corresponding schematic of its configuration.

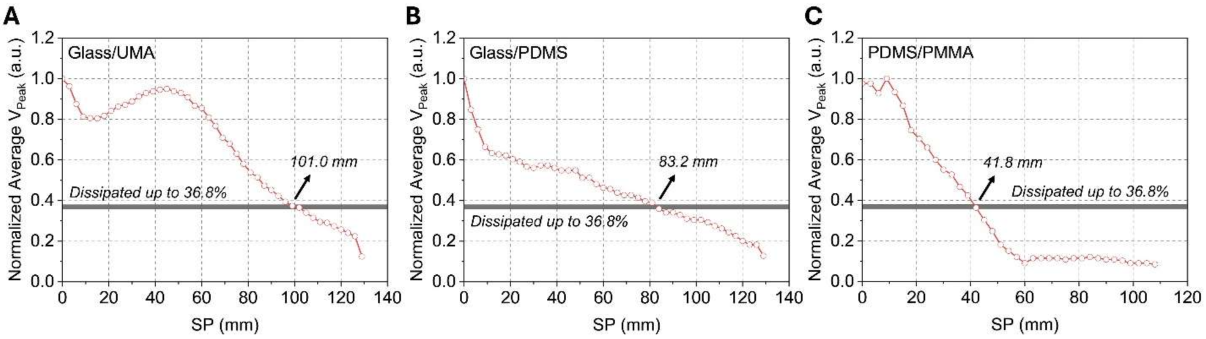


**Fig. S31**. **Normalized average voltage peak profiles of MSPC devices as a function of SP, measured after an 5-minute charging process with nitrile.** The maximum electrode spacing for the Glass/UMA, Glass/PDMS, and PDMS/PMMA devices was determined to be 101 mm, 83 mm, and 41 mm, respectively, when the profile exhibited a dissipation of 36.8%.

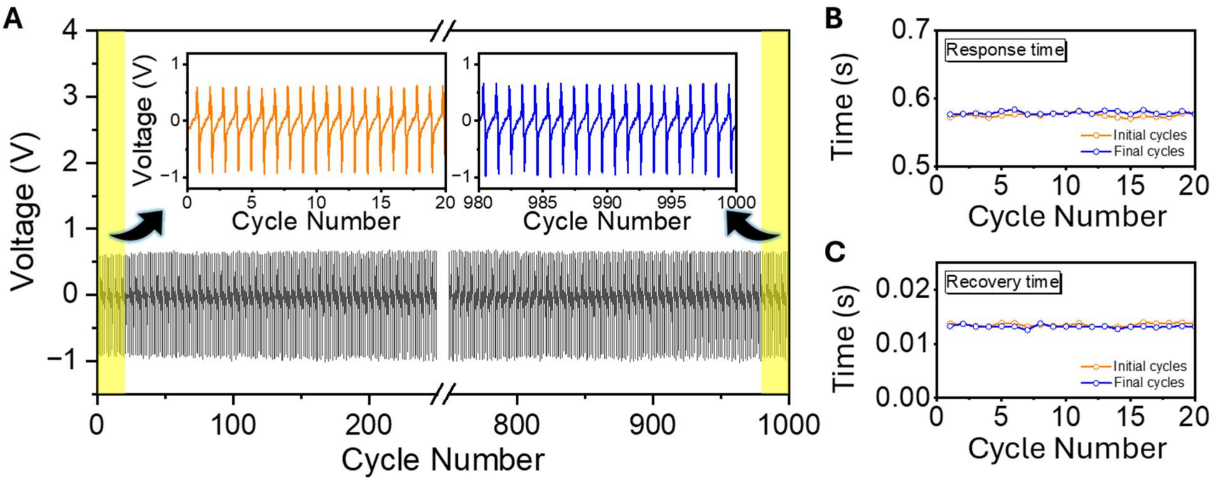


**Fig. S32. Cycling durability and temporal stability of the MSPC-based tactile sensor based on the glass/PDMS device.** (A) Voltage output recorded over 1000 repetitive stimulation cycles, with insets showing enlarged signals for the initial and final cycles. (B, C) Response and recovery times extracted from the initial and final cycles.

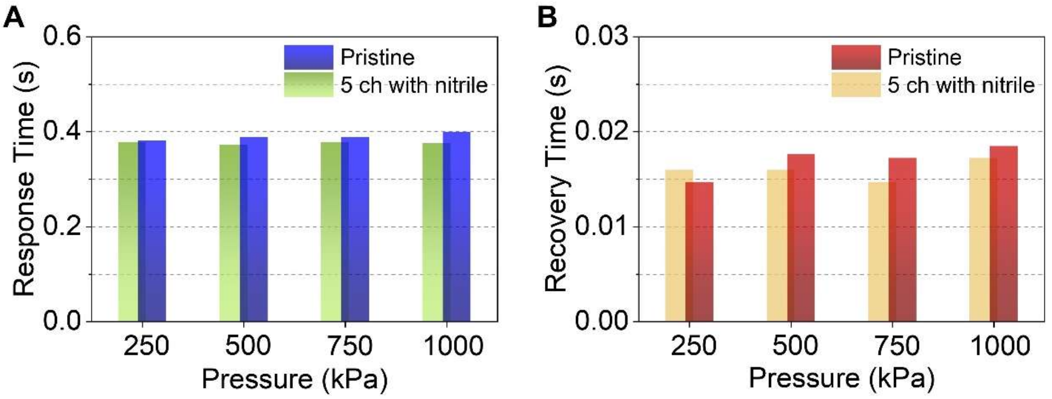


**Fig. S33. Temporal dynamics of the MSPC-based tactile sensor as a function of applied pressure.** (A) Average response time as a function of applied pressure measured in the pristine state and after 5 min nitrile charging. (B) Average recovery time as a function of applied pressure measured in the pristine state and after 5 min nitrile charging.

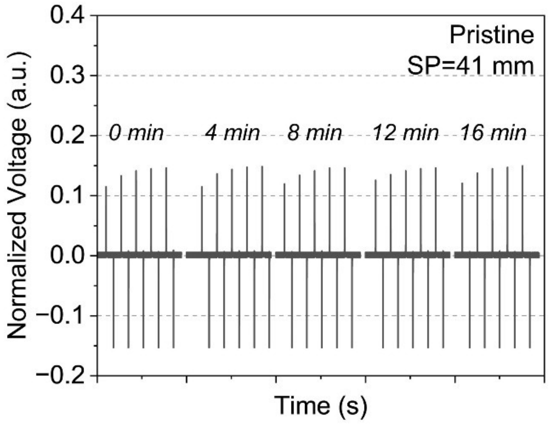


**Fig. S34**. **Time-dependent normalized voltage profiles of a pristine cell at SP = 41 mm, recorded at decay times of 0, 4, 8, 12, and 16 min.** No significant change in voltage response was observed over time, confirming the absence of MSPC channel formation under pristine conditions.

**Note S2. Strategies for improving signal-to-noise performance in MSPC sensors.**

For achieving higher sensing performance in MSPC-based tactile systems, several approaches can be adopted to enhance signal-to-noise characteristics without altering the intrinsic behavior of the MSPC channels.

i) Circuit-level optimization.

The readout electronics can be refined through the use of buffered high-impedance inputs, low-noise preamplifiers, or transimpedance amplifier stages. Such configurations can increase effective voltage gain and suppress electrical noise introduced at the circuit level.

ii) Electrode routing, grounding, and shielding.

Noise originating from environmental electromagnetic interference or parasitic coupling can be reduced by optimizing electrode layout, improving grounding schemes, and incorporating shielding layers. Differential readout between symmetric or adjacent electrodes can further reduce common-mode noise and improve measurement stability.

iii) Temporal filtering and algorithmic processing.

MSPC signals exhibit characteristic temporal profiles, such as distinct voltage peaks and predictable decay behavior, that allow for effective noise reduction through temporal filtering (e.g., low-pass filtering, moving-average smoothing). Ratio-based normalization or referencing to calibrated decay curves can additionally suppress baseline drift and enhance robustness in practical sensing environments.

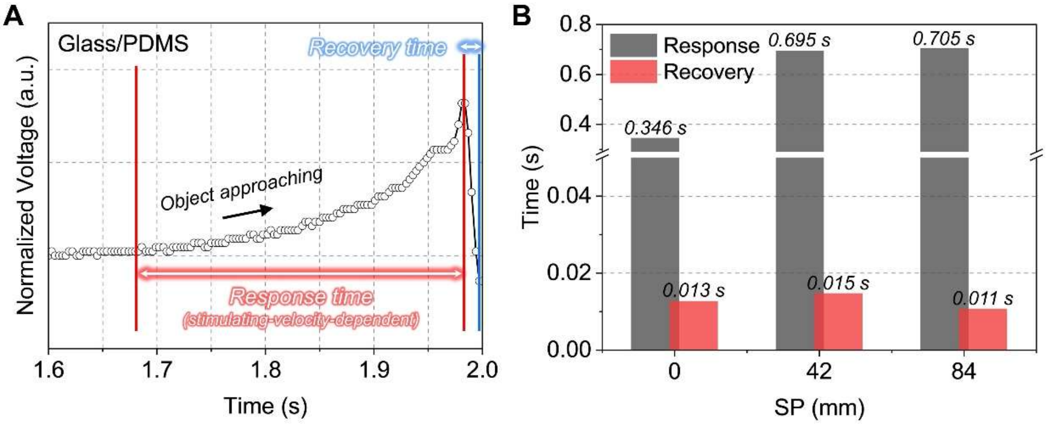


**Fig. S35. Response and recovery time characteristics of MSPC-based devices.** (A) Normalized voltage profile measured from a glass/PDMS device after 5ch with nitrile at SP = 0 mm. The plot illustrates the criteria used to define the response time (interval from object approach to the peak generation) and recovery time (interval from peak release to signal return to baseline). (B) Average response and recovery times extracted at three stimulus positions (SP = 0, 42, and 84 mm), which correspond to spacing conditions similar to those used in the time-stamping tactile sensing experiments. The response time reflects the combined effect of the approaching velocity of the stimulating object (145 mm/s) and the charge-carrier transport dynamics along the dielectric interface. Consequently, the measured response time is strongly stimulating-velocity-dependent and can be substantially reduced under faster approach conditions, whereas the recovery time remains short (<0.015 s).

**Note S3. Multi-touch considerations and potential strategies for MSPC-based sensors.**
The MSPC-based tactile sensor is inherently designed as a single-touch time-stamping system. Its spatial and temporal decoding relies on interpreting a single distributed voltage pattern measured across eight electrodes. For a single touch, this pattern uniquely reflects the four adjacent electrodes with the highest signals, the calibrated distance-dependent attenuation at SP = 41 mm and 85 mm, and the characteristic temporal decay of an MSPC channel.

When two or more touches occur simultaneously, their voltage contributions superimpose at each electrode. Because the sensor captures only eight voltage values while the number of unknowns (multiple touch positions and their respective decay states) increases, the resulting voltage distribution no longer matches any valid single-touch forward model. This leads to an underdetermined problem with no unique inverse solution, making fully concurrent multi-touch decoding unachievable in the present architecture. Sequential touches separated by more than the MSPC response time can still be decoded, because each event produces a distinct peak and decay profile that can be interpreted individually using the established single-touch mechanism.

Although multi-touch operation is beyond the scope of the current device, several engineering strategies could, in principle, enable multi-touch discrimination in future MSPC-based platforms. Increasing the spatial sampling density through additional electrodes would reduce ambiguity in reconstructing overlapping voltage patterns. Introducing orthogonal or vertically stacked dielectric layers could create partially independent propagation pathways, allowing touches at different locations to modulate different dielectric networks. Encoding mechanical inputs with distinct temporal or frequency signatures may also allow stimulus separation through time- or frequency-domain analysis. Alternatively, implementing multi-layer MSPC networks could provide parallel channels capable of separating voltage contributions from multiple simultaneous contacts.

These approaches extend beyond the purpose of the present single-touch secure time-stamping system but highlight potential directions for expanding MSPC-based spatiotemporal sensing toward multi-touch-capable architectures.

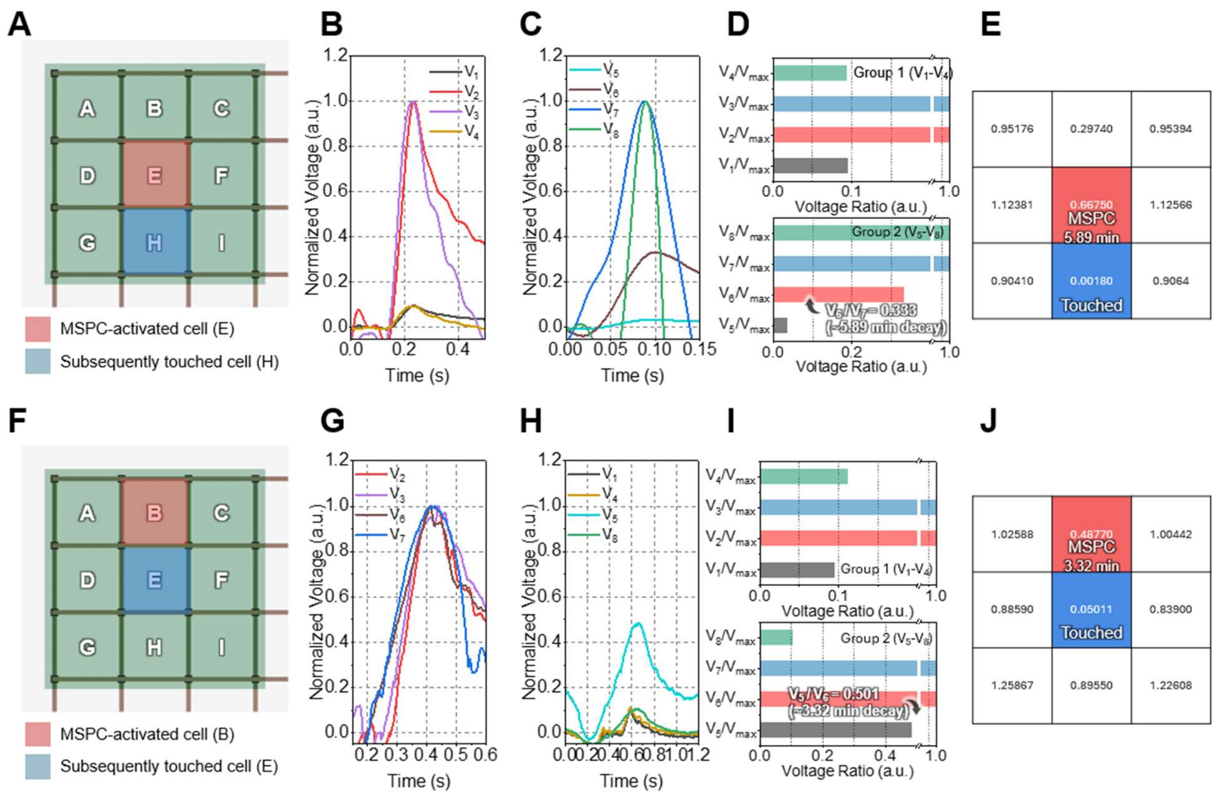

**Fig. S36. Additional spatiotemporal decoding scenarios for MSPC-based tactile sensing.** (A) Scenario in which the MSPC channel is activated at cell E, followed by a touch at cell H after 6 min. Corresponding normalized voltage profiles measured at the 8 electrodes are shown in (B-C). (D) Voltage ratios for each electrode normalized by the maximum value within each set, where the MSPC-mediated pathway yields an elevated ratio (V6/V7 = 0.333) used to infer the elapsed time from the calibrated decay behavior. (E) Spatial mapping of the voltage asymmetry metric M, indicating the MSPC-activated cell E and the subsequently touched cell H. (F) Scenario in which the MSPC channel is activated at cell B, followed by a touch at cell E after 3 min. Corresponding normalized voltage profiles measured at the 8 electrodes are shown in (G-H). (I) Voltage ratios normalized within each set, where the elevated pathway ratio (V5/V6 = 0.501) is used to estimate the elapsed time from the calibrated decay behavior. (J) Spatial mapping of the voltage asymmetry metric M, identifying the MSPC-activated cell B and the subsequently touched cell E.

**Note S4. Additional test scenarios for strengthening time error assessment.**

To strengthen the reliability of the time error analysis, two additional tests were examined: i) MSPC activation at cell E followed by a touch at cell H, and ii) MSPC activation at cell B followed by a touch at cell E.

In the first added scenario, the MSPC was activated at cell E, and cell H was touched after 6 min (**Fig. S36A**). Upon touching cell H, the adjacent electrodes $V_2$, $V_3$, $V_6$, and $V_7$ exhibited the highest normalized responses of approximately 1.00, confirming correct spatial localization (**Figs. S36B–36C**). The electrodes one cell away from H at SP = 41 mm showed attenuated signals consistent with the calibrated dissipation, with $V_1$ = 0.096 and $V_4$ = 0.094. Importantly, electrodes located along the MSPC-active pathway displayed elevated responses relative to the pristine attenuation model: $V_6$ reached 0.3325 despite being separated by one cell, and $V_5$ exhibited 0.035 even though it corresponds to an approximately two-cell separation of ~85 mm. Using the voltage-ratio-based calibration, the extracted ratio $V_6/V_7$ = 0.333 yielded an inferred delay of 5.89 min, corresponding to an absolute deviation of 0.11 min and a time error of 1.8% (**Fig. S36D**). State classification was further supported by the voltage asymmetry metric M, which yielded M = 0.00180 for the touched cell and M = 0.66750 for the MSPC-activated cell (**Fig. S36E**).

In the second added scenario, the MSPC was activated at cell B, and cell E was touched after 3 min (**Fig. S36F**). The adjacent electrodes $V_2$, $V_3$, $V_6$, and $V_7$ again exhibited the highest responses, consistent with the deterministic SP-based spatial readout (**Fig. S36G**). The electrodes at SP = 41 mm from cell E displayed attenuated responses, with $V_1$ = 0.095, $V_4$ = 0.112, and $V_8$ = 0.105 (**Fig. S36H**). In contrast, the electrode response along the MSPC-active pathway remained elevated: V5 reached 0.483 despite being at SP = 41 mm, consistent with signal propagation through the MSPC-activated cell. The ratio $V_5/V_6$ = 0.501 corresponded to an inferred delay of 3.32 min from the Boltzmann calibration, resulting in a time error of 11% (**Fig. S36I**). The voltage asymmetry metric further supported this interpretation, yielding M = 0.05011 for the touched cell E and M = 0.48770 for the MSPC-activated cell B (**Fig. S36J**).

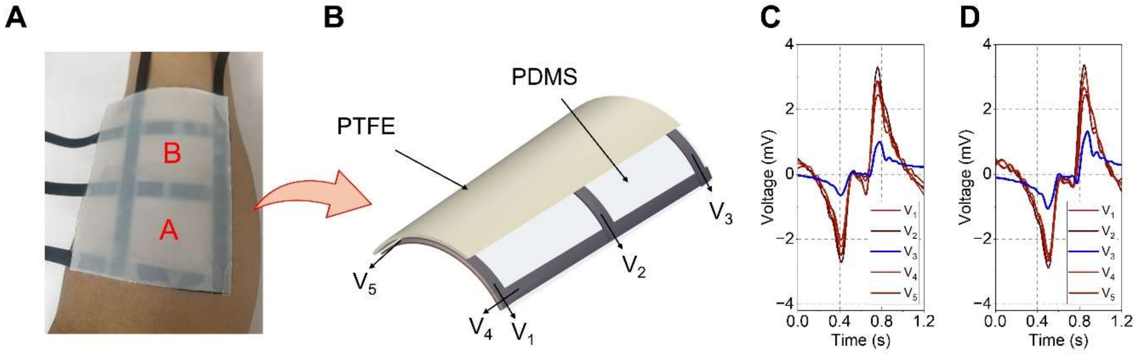


**Fig. S37. Proof-of-concept electronic-skin (e-skin) demonstration using a PTFE/PDMS MSPC device.** (A) Photograph of a flexible two-cell e-skin device (cells A and B) mounted on a forearm. (B) Schematic illustration of the PTFE/PDMS layered structure and the five-electrode readout configuration ($V_1$–$V_5$) used to record voltage signals from the device. (C) Voltage signals measured upon stimulating cell A in the pristine state, showing higher responses at electrodes adjacent to the stimulated region ($V_1$, $V_2$, $V_4$, $V_5$) and the significantly attenuated response at the more distant electrode $V_3$. (D) Voltage signals measured after repeated hand-touch activation (≈500 touches), where the response at $V_3$ increases, indicating enhanced long-range signal transmission enabled by MSPC formation in the flexible dielectric-based e-skin format.

**Note S5. Wearable electronic-skin demonstration based on the PTFE/PDMS MSPC device.**

To demonstrate application-level feasibility in a wearable format, a proof-of-concept e-skin patch was constructed using the PTFE/PDMS dielectric pair (**Fig. S37A**). The device comprises two sensing cells (A, B) and five bottom electrodes ($V_1$–$V_5$) (**Fig. S37B**).

Before MSPC activation, localized stimulation at cell A produced comparable voltage responses at nearby electrodes ($V_1$, $V_2$, $V_4$, $V_5$), while the distant electrode $V_3$ showed clear attenuation (**Fig. S37C**). After conditioning by repeated tapping (~500 touches) to induce MSPC formation, stimulation at the same location led to a pronounced increase in the $V_3$ signal (**Fig. S37D**), despite unchanged geometry, indicating enhanced long-range signal transmission.

Because the spatial voltage distribution depends on the MSPC activation state, which exhibits time-dependent decay, this wearable configuration preserves the time-stamping functionality demonstrated in the planar device. These results suggest that MSPC-enabled signal propagation can relax geometric attenuation constraints in scalable dielectric-based e-skin systems.

**Table S1. Comparison of encoded information, cycling durability, and response time between MSPC-based tactile sensing and representative conventional tactile sensor mechanisms.**

| | Encoded information | Cycle durability | Response time [ms] | Source |
|---|---|---|---|---|
| MSPC | Spatio-temporal | >1000 | 346 (at SP = 0) | *This study* |
| Piezoelectric | Spatial | 10000 | 100 | *Zhen et al., 2024 (64)* |
| | Spatial | 80000 | 10 | *Lin et al., 2021 (65)* |
| Piezoresistive | Spatial | >10000 | 118 | *Liu et al., 2025 (66)* |
| | Spatial | 3735 | 50 | *Zhao et al., 2025 (67)* |
| Triboelectric | Spatial | 5000 | 80 | *Lin et al., 2018 (68)* |
| | Spatial | 3600 | 80 | *Zhu et al., 2024 (69)* |
| | Spatial | 5000 | 46 | *Wang et al., 2025 (70)* |
| Capacitive | Spatial | 500 | 9 | *Liu et al., 2025 (71)* |
| | Spatial | 100 | 32 | *Yang et al., 2024 (72)* |

**REFERENCES**


1. J. Qu, B. Mao, Z. Li, Y. Xu, K. Zhou, X. Cao, Q. Fan, M. Xu, B. Liang, H. Liu, X. Wang, X. Wang, Recent progress in advanced tactile sensing technologies for soft grippers. *Adv. Funct. Mater.* **33**, 2306249 (2023).

2. S. Li, X. Chen, X. Li, H. Tian, C. Wang, B. Nie, J. He, J. Shao, Bioinspired robot skin with mechanically gated electron channels for sliding tactile perception. *Sci. Adv.* **8**, eade0720 (2022).

3. D. Kong, Y. Lu, S. Zhou, M. Wang, G. Pang, B. Wang, L. Chen, X. Huang, H. Lyu, K. Xu, Super-resolution tactile sensor arrays with sparse units enabled by deep learning. *Sci. Adv.* **11**, eadv2124 (2025).

4. S. Chun, J.-S. Kim, Y. Yoo, Y. Choi, S. J. Jung, D. Jang, G. Lee, K.-I. Song, K. S. Nam, I. Youn, D. Son, C. Pang, Y. Jeong, H. Jung, Y.-J. Kim, B.-D. Choi, J. Kim, S.-P. Kim, W. Park, S. Park, An artificial neural tactile sensing system. *Nat. Electron.* **4**, 429–438 (2021).

5. Z. Hu, L. Lin, W. Lin, Y. Xu, X. Xia, Z. Peng, Z. Sun, Z. Wang, Machine learning for tactile perception: Advancements, challenges, and opportunities. *Adv. Intell. Syst.* **5**, 2200371 (2023).

6. B. Seo, Y. Cha, Y. Choi, S. Kim, W. Choi, Rationally designed micropixelation-free tactile sensors via contour profile of triboelectric field propagation. *Nano Energy* **109**, 108255 (2023).

7. S. He, J. Dai, D. Wan, S. Sun, X. Yang, X. Xia, Y. Zi, Biomimetic bimodal haptic perception using triboelectric effect. *Sci. Adv.* **10**, eado6793 (2024).

8. B. Ramasubramanian, S. Sundarrajan, R. P. Rao, M. Reddy, V. Chellappan, S. Ramakrishna, Novel low-carbon energy solutions for powering emerging wearables, smart textiles, and medical devices. *Energ. Environ. Sci.* **15**, 4928–4981 (2022).

9. S. Zhang, Y. Yang, Y. Sun, N. Liu, F. Sun, B. Fang, Artificial skin based on visuo-tactile sensing for 3D shape reconstruction: Material, method, and evaluation. *Adv. Funct. Mater.* **35**, 2411686 (2025).

10. A. Babu, I. Aazem, R. Walden, S. Bairagi, D. M. Mulvihill, S. C. Pillai, Electrospun nanofiber based TENGs for wearable electronics and self-powered sensing. *Chem. Eng. J.* **452**, 139060 (2023).

11. M. Ji, Z. Wang, J. Wu, L. Huang, M. Zheng, G. Cheng, H. Cai, J. Luo, H. Zhou, Z. L. Wang, Machine learning–assisted triboelectric nanogenerator technology for intelligent sports. *Sci. Adv.* **11**, eadz3515 (2025).

12. W. Peng, R. Zhu, Q. Ni, J. Zhao, X. Zhu, Q. Mei, C. Zhang, L. Liao, Functional tactile sensor based on arrayed triboelectric nanogenerators. *Adv. Energy Mater.* **14**, 2403289 (2024).

13. S.-Z. Liu, W.-T. Guo, H. Chen, Z.-X. Yin, X.-G. Tang, Q.-J. Sun, Recent progress on flexible self-powered tactile sensing platforms for health monitoring and robotics. *Small* **20**, e2405520 (2024).

14. G. Wu, X. Li, R. Bao, C. Pan, Innovations in tactile sensing: Microstructural designs for superior flexible sensor performance. *Adv. Funct. Mater.* **34**, 2405722 (2024).

15. B. Wu, T. Jiang, Z. Yu, Q. Zhou, J. Jiao, M. L. Jin, Proximity sensing electronic skin: Principles, characteristics, and applications. *Adv. Sci.* **11**, e2308560 (2024).

16. R. Qin, J. Nong, K. Wang, Y. Liu, S. Zhou, M. Hu, H. Zhao, G. Shan, Recent advances in flexible pressure sensors based on MXene materials. *Adv. Mater.* **36**, e2312761 (2024).

17. S. Mishra, S. Mohanty, A. Ramadoss, Functionality of flexible pressure sensors in cardiovascular health monitoring: A review. *ACS Sens.* **7**, 2495–2520 (2022).

18. R. L. Bulathsinghala, W. Ding, R. D. I. G. Dharmasena, Triboelectric nanogenerators for wearable sensing applications: A system level analysis. *Nano Energy* **116**, 108792 (2023).

19. B. Seo, Y. Cha, S. Kim, W. Choi, Rational design for optimizing hybrid thermo-triboelectric generators targeting human activities. *ACS Energy Lett.* **4**, 2069–2074 (2019).

20. H.-J. Yoon, S.-W. Kim, Nanogenerators to power implantable medical systems. *Joule* **4**, 1398–1407 (2020).

21. Y. Dai, H. Hu, M. Wang, J. Xu, S. Wang, Stretchable transistors and functional circuits for human-integrated electronics. *Nat. Electron.* **4**, 17–29 (2021).

22. J. Wang, S. Xu, C. Zhang, A. Yin, M. Sun, H. Yang, C. Hu, H. Liu, Field effect transistor-based tactile sensors: From sensor configurations to advanced applications. *InfoMat* **5**, e12376 (2023).

23. O. Ozioko, R. Dahiya, Smart tactile gloves for haptic interaction, communication, and rehabilitation. *Adv. Intell. Syst.* **4**, 2100091 (2022).

24. J. Park, Y. Lee, S. Cho, A. Choe, J. Yeom, Y. G. Ro, J. Kim, D.-h. Kang, S. Lee, H. Ko, Soft sensors and actuators for wearable human–machine interfaces. *Chem. Rev.* **124**, 1464–1534 (2024).

25. Y. Huang, K. Yao, J. Li, D. Li, H. Jia, Y. Liu, C. K. Yiu, W. Park, X. Yu, Recent advances in multi-mode haptic feedback technologies towards wearable interfaces. *Mater. Today Phys.* **22**, 100602 (2022).

26. S. Duan, H. Zhang, L. Liu, Y. Lin, F. Zhao, P. Chen, S. Cao, K. Zhou, C. Gao, Z. Liu, Q. Shi, C. Lee, J. Wu, A comprehensive review on triboelectric sensors and AI-integrated systems. *Mater. Today* **80**, 450–480 (2024).

27. G. Mu, Y. Zhang, Z. Yan, Q. Yu, Q. Wang, Recent advancements in wearable sensors: Integration with machine learning for human–machine interaction. *RSC Adv.* **15**, 7844–7854 (2025).

28. B. Seo, Y. Choi, D. Noh, J. Kim, X. Chen, W. Choi, Triangular electrode arrangement for minimizing electrode density and footprint in tactile sensors and flexible electronics. *Device* **3**, 100602 (2025).

29. R. Bagherzadeh, S. Abrishami, A. Shirali, A. R. Rajabzadeh, Wearable and flexible electrodes in nanogenerators for energy harvesting, tactile sensors, and electronic textiles: Novel materials, recent advances, and future perspectives. *Mater. Today Sustain.* **20**, 100233 (2022).

30. Q. Shu, Y. Pang, Q. Li, Y. Gu, Z. Liu, B. Liu, J. Li, Y. Li, Flexible resistive tactile pressure sensors. *J. Mater. Chem. A* **12**, 9296–9321 (2024).

31. X. Xiao, J. Yin, J. Xu, T. Tat, J. Chen, Advances in machine learning for wearable sensors. *ACS Nano* **18**, 22734–22751 (2024).

32. H. Fang, J. Guo, H. Wu, Wearable triboelectric devices for haptic perception and VR/AR applications. *Nano Energy* **96**, 107112 (2022).

33. K. Kim, H. Yang, J. Lee, W. G. Lee, Metaverse wearables for immersive digital healthcare: A review. *Adv. Sci.* **10**, e2303234 (2023).

34. Q. Jiang, M. F. Antwi-Afari, S. Fadaie, H.-Y. Mi, S. Anwer, J. Liu, Self-powered wearable Internet of Things sensors for human-machine interfaces: A systematic literature review and science mapping analysis. *Nano Energy* **131**, 110252 (2024).

35. W. Tang, Q. Sun, Z. L. Wang, Self-powered sensing in wearable electronics—A paradigm shift technology. *Chem. Rev.* **123**, 12105–12134 (2023).

36. D. Yan, J. Wang, J. Xiang, Y. Xing, L.-H. Shao, A flexoelectricity-enabled ultrahigh piezoelectric effect of a polymeric composite foam as a strain-gradient electric generator. *Sci. Adv.* **9**, eadc8845 (2023).

37. A. Libanori, G. Chen, X. Zhao, Y. Zhou, J. Chen, Smart textiles for personalized healthcare. *Nat. Electron.* **5**, 142–156 (2022).

38. B. Seo, Y. Cha, S. Kim, W. Choi, Tunable current duration in triboelectric generators via capacitive air gaps. *Int. J. Energy Res.* **45**, 5619–5628 (2021).

39. F. Yin, H. Niu, E.-S. Kim, Y. K. Shin, Y. Li, N.-Y. Kim, Advanced polymer materials-based electronic skins for tactile and non-contact sensing applications. *InfoMat* **5**, e12424 (2023).

40. J. Kim, Y. Choi, H. Jang, S. Jiong, X. Chen, B. Seo, W. Choi, Thermo-chemo-mechanically robust, multifunctional MXene/PVA/PAA-Hanji textile with energy harvesting, EMI shielding, flame-retardant, and joule heating capabilities. *Adv. Mater.* **36**, e2411248 (2024).

41. A. Baburaj, S. Banerjee, A. K. Aliyana, C. Shee, M. Banakar, S. Bairagi, S. K. Naveen Kumar, S. W. Ali, G. K. Stylios, Biodegradable based TENGs for self-sustaining implantable medical devices. *Nano Energy* **127**, 109785 (2024).

42. B. Seo, H. Han, K. Kim, D. Noh, J. H. Shim, W. Choi, Humidity-thermoelectric bimodal energy harvester for sustainable power generation. *Nano Energy* **107**, 108120 (2023).

43. C. Dong, A. Leber, D. Yan, H. Banerjee, S. Laperrousaz, T. Das Gupta, S. Shadman, P. M. Reis, F. Sorin, 3D stretchable and self-encapsulated multimaterial triboelectric fibers. *Sci. Adv.* **8**, eabo0869 (2022).

44. Y. Kamiyama, R. Tamate, T. Hiroi, S. Samitsu, K. Fujii, T. Ueki, Highly stretchable and self-healable polymer gels from physical entanglements of ultrahigh–molecular weight polymers. *Sci. Adv.* **8**, eadd0226 (2022).

45. X. Xiong, J. Liang, W. Wu, Principle and recent progress of triboelectric pressure sensors for wearable applications. *Nano Energy* **113**, 108542 (2023).

46. X. Xie, Y. Fang, C. Lu, Y. Tao, L. Yin, Y. Zhang, Z. Wang, S. Wang, J. Zhao, X. Tu, X. Sun, E. G. Lim, C. Zhao, Y. Liu, Z. Wen, Effective interfacial energy band engineering strategy toward high-performance triboelectric nanogenerator. *Chem. Eng. J.* **452**, 139469 (2023).

47. H. P. Palani Velayuda Shanmugasundram, E. Jayamani, K. H. Soon, A comprehensive review on dielectric composites: Classification of dielectric composites. *Renew. Sustain. Energy Rev.* **157**, 112075 (2022).

48. Y. Choi, J. Kim, J. Lee, X. Chen, B. Seo, W. Choi, Recent progress on 2D-material-based smart textiles: Materials, methods, and multifunctionality. *Adv. Eng. Mater.* **27**, 2500188 (2025).

49. J. Xu, J. Pan, T. Cui, S. Zhang, Y. Yang, T.-L. Ren, Recent progress of tactile and force sensors for human–machine interaction. *Sensors* **23**, 1868 (2023).

50. S. Yu, T. H. Park, W. Jiang, S. W. Lee, E. H. Kim, S. Lee, J.-E. Park, C. Park, Soft human–machine interface sensing displays: Materials and devices. *Adv. Mater.* **35**, 2204964 (2023).

51. S. Pyo, J. Lee, K. Bae, S. Sim, J. Kim, Recent progress in flexible tactile sensors for human-interactive systems: From sensors to advanced applications. *Adv. Mater.* **33**, e2005902 (2021).

52. J. Li, Z. Fang, D. Wei, Y. Liu, Flexible pressure, humidity, and temperature sensors for human health monitoring. *Adv. Healthc. Mater.* **13**, e2401532 (2024).

53. D. Geng, K. Wang, L. Li, K. Myny, A. Nathan, J. Jang, Y. Kuo, M. Liu, Thin-film transistors for large-area electronics. *Nat. Electron.* **6**, 963–972 (2023).

54. Q.-K. Feng, S.-L. Zhong, J.-Y. Pei, Y. Zhao, D.-L. Zhang, D.-F. Liu, Y.-X. Zhang, Z.-M. Dang, Recent progress and future prospects on all-organic polymer dielectrics for energy storage capacitors. *Chem. Rev.* **122**, 3820–3878 (2022).

55. D. Guo, P. Guo, L. Ren, Y. Yao, W. Wang, M. Jia, Y. Wang, L. Wang, Z. L. Wang, J. Zhai, Silicon flexoelectronic transistors. *Sci. Adv.* **9**, eadd3310 (2023).

56. W.-G. Kim, D.-W. Kim, I.-W. Tcho, J.-K. Kim, M.-S. Kim, Y.-K. Choi, Triboelectric nanogenerator: Structure, mechanism, and applications. *ACS Nano* **15**, 258–287 (2021).

57. R. Zhang, H. Olin, Material choices for triboelectric nanogenerators: A critical review. *EcoMat* **2**, e12062 (2020).

58. Y. Song, N. Wang, Y. Wang, R. Zhang, H. Olin, Y. Yang, Direct current triboelectric nanogenerators. *Adv. Energy Mater.* **10**, 2002756 (2020).

59. B. Seo, D. Noh, Y. Choi, X. Chen, R. Hu, W. Choi, Mechanical-stimuli-driven pseudo-conductive channels along dielectric heterojunction interfaces for mechanoelectric energy conversion and transmission. *Adv. Mater.* **37**, e2416952 (2025).

60. G. Shao, Work function and electron affinity of semiconductors: Doping effect and complication due to fermi level pinning. *Energy Environ. Mater.* **4**, 273–276 (2021).

61. C. Lohaus, A. Klein, W. Jaegermann, Limitation of Fermi level shifts by polaron defect states in hematite photoelectrodes. *Nat. Commun.* **9**, 4309 (2018).

62. S. R. Cowan, A. Roy, A. J. Heeger, Recombination in polymer-fullerene bulk heterojunction solar cells. *Phys. Rev. B* **82**, 245207 (2010).

63. K. Wang, C. Han, J. Li, J. Qiu, J. Sunarso, S. Liu, The mechanism of piezocatalysis: Energy band theory or screening charge effect? *Angew. Chem. Int. Ed.* **61**, e202110429 (2022).

64. L. Zhen, M. Cui, X. Bai, J. Jiang, X. Ma, M. Wang, J. Liu, B. Yang, Thin, flexible hybrid-structured piezoelectric sensor array with enhanced resolution and sensitivity. *Nano Energy* **131**, 110188 (2024).

65. W. Lin, B. Wang, G. Peng, Y. Shan, H. Hu, Z. Yang, Skin-inspired piezoelectric tactile sensor array with crosstalk-free row+column electrodes for spatiotemporally distinguishing diverse stimuli. *Adv. Sci.* **8**, 2002817 (2021).

66. Y. Liu, H. Xiao, D. Pang, S. Sun, Z. Sun, S. Liu, Hybrid structured wearable flexible piezoresistive sensor with high sensitivity and wide detection range. *Sens. Actuators A. Phys.* **388**, 116520 (2025).

67. W. Zhao, L. Geng, J. Li, H. Zhang, S. Zhang, Y. Guo, S. Cheng, W. Zhao, Flexible piezoresistive sensing device with integrated design and fabrication for health monitoring. *Chem. Eng. J.* **523**, 168600 (2025).

68. Z. Lin, J. Yang, X. Li, Y. Wu, W. Wei, J. Liu, J. Chen, J. Yang, Large-scale and washable smart textiles based on triboelectric nanogenerator arrays for self-powered sleeping monitoring. *Adv. Funct. Mater.* **28**, 1704112 (2018).

69. L. Zhu, P. Xu, B. Chang, J. Ning, T. Yan, Z. Yang, H. Lu, Hierarchical structure by self-sedimentation of liquid metal for flexible sensor integrating pressure detection and triboelectric nanogenerator. *Adv. Funct. Mater.* **34**, 2400363 (2024).

70. C. Wang, H. Niu, G. Shen, Y. Li, Self-healing hydrogel-based triboelectric nanogenerator in smart glove system for integrated drone safety protection and motion control. *Adv. Funct. Mater.* **35**, 2419809 (2025).

71. J. Liu, H. Liu, H. Guo, L. Huang, T. Lu, Self-powered iontronic capacitive sensing unit with high sensitivity in charge-output mode. *Adv. Funct. Mater.* **35**, 2412377 (2025).

72. D. Yang, K. Zhao, R. Yang, S.-W. Zhou, M. Chen, H. Tian, D.-H. Qu, A rational design of bio-derived disulfide CANs for wearable capacitive pressure sensor. *Adv. Mater.* **36**, e2403880 (2024).
